# Time Spent Outdoors Partly Accounts for the Effect of Education on Myopia

**DOI:** 10.1167/iovs.64.14.38

**Published:** 2023-11-27

**Authors:** Rosie Clark, Sander C. M. Kneepkens, Denis Plotnikov, Rupal L. Shah, Yu Huang, J. Willem L. Tideman, Caroline C. W. Klaver, Denize Atan, Cathy Williams, Jeremy A. Guggenheim, for the UK Biobank Eye and Vision Consortium

**Affiliations:** 1 School of Optometry and Vision Sciences, Cardiff University, Cardiff, United Kingdom; 2 Department of Ophthalmology, Erasmus University Medical Center, CA Rotterdam, The Netherlands; 3 Department of Epidemiology, Erasmus University Medical Center, CA Rotterdam, The Netherlands; 4 Generation R Study Group, Erasmus University Medical Center, CA Rotterdam, The Netherlands; 5 Central Research Laboratory, Kazan State Medical University, Kazan, Russia; 6 Population Health Sciences Institute, Faculty of Medical Sciences, Newcastle University, International Centre for Life, Central Parkway, Newcastle upon Tyne, United Kingdom; 7 Guangdong Eye Institute, Department of Ophthalmology, Guangdong Provincial People's Hospital, Guangdong Academy of Medical Sciences, Guangzhou, China; 8 Department of Ophthalmology, Martini Hospital, RM Groningen, The Netherlands; 9 Institute of Molecular and Clinical Ophthalmology, Basel, Switzerland; 10 Department of Ophthalmology, Radboud University Medical Center, GA Nijmegen, The Netherlands; 11 Translational Health Sciences, Bristol Medical School, University of Bristol, Bristol, BS81NU, United Kingdom; 12 Centre for Academic Child Health, Population Health Sciences, Bristol Medical School, University of Bristol, Bristol, BS81NU, United Kingdom

**Keywords:** myopia, time outdoors, educational attainment, refractive error, Mendelian randomization

## Abstract

**Purpose:**

The purpose of this study was to investigate if education contributes to the risk of myopia because educational activities typically occur indoors or because of other factors, such as prolonged near viewing.

**Methods:**

This was a two-sample Mendelian randomization study. Participants were from the UK Biobank, Avon Longitudinal Study of Parents and Children, and Generation R. Genetic variants associated with years spent in education or time spent outdoors were used as instrumental variables. The main outcome measures were: (1) spherical equivalent refractive error attained by adulthood, and (2) risk of an early age-of-onset of spectacle wear (EAOSW), defined as an age-of-onset of 15 years or below.

**Results:**

Time spent outdoors was found to have a small genetic component (heritability 9.8%) that tracked from childhood to adulthood. A polygenic score for time outdoors was associated with children's time outdoors; a polygenic score for years spent in education was inversely associated with children's time outdoors. Accounting for the relationship between time spent outdoors and myopia in a multivariable Mendelian randomization analysis reduced the size of the causal effect of more years in education on myopia to −0.17 diopters (D) per additional year of formal education (95% confidence interval [CI] = −0.32 to −0.01) compared with the estimate from a univariable Mendelian randomization analysis of −0.27 D per year (95% CI = −0.41 to −0.13). Comparable results were obtained for the outcome EAOSW.

**Conclusions:**

Accounting for the effects of time outdoors reduced the estimated causal effect of education on myopia by 40%. These results suggest about half of the relationship between education and myopia may be mediated by children not being outdoors during schooling.

Myopia affects about 30% of the population in Europe and the United States, and more than 50% of people in parts of East and Southeast Asia.^1^ Globally, uncorrected myopia is a major source of sight impairment and adversely affects work and quality of life.^2^ Although correction of myopia with spectacles, contact lenses, or refractive surgery provides clear distance vision, it does not reduce the risk of sight-threatening pathologies that are associated with axial elongation in myopic eyes.^3^ In particular, the risk of retinal detachment and myopic macular degeneration (MMD) increase exponentially with the degree of myopia.^4^ In 2020, the worldwide prevalence of MMD was estimated to be 2.1%, 95% confidence interval (CI) = 1.3 to 3.3%, with the prevalence in East and Southeast Asia almost twice as high as in Western countries.^5^ The direct costs, globally, for eye examinations, spectacles, and treatment for myopia-related eye disorders was estimated at US $359 billion in 2019.^6^ The prevalence of myopia is predicted to increase 7-fold between 2000 and 2050, such that myopia will become a leading cause of permanent blindness worldwide.^7^ Treatments to slow the progression of myopia are available, however, currently, their long-term efficacy is limited to about 1 diopter (D), on average, and their cost is prohibitively expensive for many families.^8^^–^^10^

A range of genetic and lifestyle risk factors for myopia have been identified.^3^ Among lifestyle risk factors, there is strong evidence that myopia is causally related to more years spent in education and less time outdoors during childhood.^1^ Randomized controlled trials (RCTs) have shown that children assigned to an additional 40 to 80 minutes outdoors each school day had a reduced incidence of myopia (4–9% reduction in absolute terms, and 23-50% reduction in relative terms).^11^^–^^13^ RCTs to assess the impact of time spent in education on myopia would be unethical. Instead, evidence for a causal effect of years of education on myopia has been obtained using two alternative study designs: Mendelian randomization^14^^–^^16^ and regression discontinuity.^17^^–^^19^ These two quasi-experimental methods stratify individuals into groups with differing levels of exposure to the risk factor of interest. In Mendelian randomization, the random assortment of genetic variants at conception (single nucleotide polymorphisms [SNPs]) that predispose individuals to higher versus lower levels of exposure to the risk factor acts analogously to the random assignment of participants to the treatment versus control arm of an RCT. For example, in the previous Mendelian randomization studies of the relationship between education and myopia, the investigators evaluated the refractive error of individuals who inherited SNPs that predisposed them to higher versus fewer years of schooling.^14^^–^^16^ Advantages of Mendelian randomization compared to observational studies of an exposure-outcome relationship are that Mendelian randomization is robust to reverse causation and subject to different sources of bias from confounders, such as socio-economic status (SES).

In Taiwan, the implementation of a policy program “Tian-Tian Outdoor 120” encouraging children to spend 120 minutes per school day in outdoor activities coincided with the reversal of a long-term trend of increasing myopia prevalence.^20^ Given that education typically takes place indoors, an important gap in existing knowledge is the extent to which the causal association between years spent in education and myopia is mediated by spending time indoors in the classroom versus by other aspects of schooling, such as long periods of near viewing. We applied a multivariable Mendelian randomization (MVMR) analysis to address this question. Figure 1 provides an overview of the design of the current work. Briefly, summary statistics from genomewide association studies (GWAS) were obtained for three traits measured in nonoverlapping samples of adults: time spent outdoors, years spent in education, and refractive error. Next, the GWAS variants associated with time spent outdoors and years spent in education were validated as being associated with the time children spent outdoors and reading, respectively (it was already known that variants obtained in a GWAS for refractive error in adulthood are associated with refractive error development in childhood).^21^^–^^24^ Finally, the three sets of GWAS summary statistics were applied in Mendelian randomization analyses to explore the causal relationship among education, time outdoors, and refractive error.

**Figure 1.**
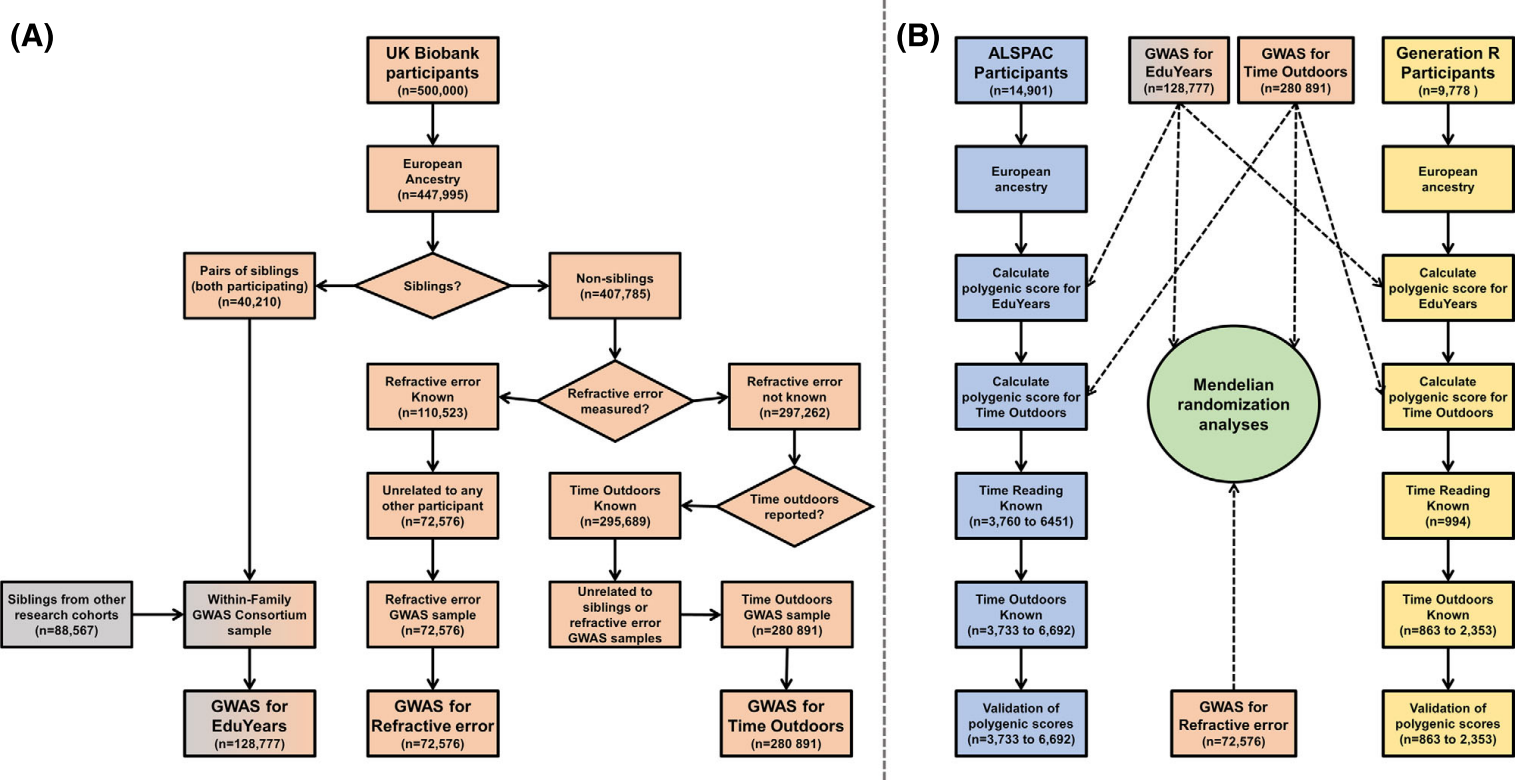
**Outline of the study design.** Panel (**A**) The study made use of three sets of GWAS summary statistics: a GWAS for time spent outdoors, a GWAS for spherical equivalent refractive error and a GWAS for years spent in education (EduYears). The three GWAS analyses were performed in nonoverlapping samples. The GWAS for spherical equivalent refractive error and the GWAS for time outdoors were newly performed analyses carried out in the current work, using samples from the UK Biobank. The GWAS for EduYears was performed in earlier work reported by the Within Family GWAS Consortium^33^; the GWAS for EduYears included participants from UK Biobank and other cohorts (HUNT, Minnesota Twins, MoBa, Netherlands Twin Register, ORCADES, QIMR Berghofer Medical Research Institute, Swedish Twin Registry I, II, and YATSS, TEDS, TwinsUK, and Viking). Panel (**B**) Two child cohorts, ALSPAC and Generation R, were used to validate that (i) the GWAS for time outdoors identified genetic variants associated with the time children spent outdoors, and (ii) the GWAS for EduYears identified genetic variants associated with the time children spent reading. The Mendelian randomization analyses were performed using only the GWAS summary statistics for Time Outdoors, EduYears, and spherical equivalent refractive error. (Note that a GWAS for an early age-of-onset of spectacle wear [EAOSW] was also performed; this was done using the same sample as the GWAS for spherical equivalent refractive error. The summary statistics from the GWAS for EAOSW were used in place of those from the GWAS for spherical equivalent refractive error in a separate set of Mendelian randomization analyses). The sample size for the ALSPAC and Generation R analyses varied depending on the number of questionnaire respondents at each age, as detailed in Supplementary Tables S2 and S3.

## Methods

### Study Cohorts

We analyzed data from three cohorts: the UK Biobank, the Avon Longitudinal Study of Parents and Children (ALSPAC) study, and the Generation R study. The UK Biobank is a longitudinal study of approximately 500,000 adults aged 40 to 70 years, who were recruited between January 2006 and October 2010.^25^ Participants attended a baseline visit at one of 22 assessment centers across England, Scotland, and Wales. Genotyping of participants was reported by Bycroft et al.^26^ Ethics approval for the study was obtained from the Northwest Multi-center Research Ethics Committee (Reference: 11/NW/0382). Participants provided informed consent and were free to withdraw from the study at any time. The research adhered to the tenets of the Declaration of Helsinki. The ALSPAC study^27^^,^^28^ recruited pregnant women resident in Avon, England, with expected dates of delivery April 1991 to December 1992. After the age of 7 years, attempts were made to bolster the initial sample with eligible cases who had failed to join the study originally, which resulted in an additional 913 children being enrolled. The total sample size for children alive at 1 year of age was 14,901, along with their mothers or guardians. The study website contains details of all the data that are available through a fully searchable data dictionary and variable search tool: http://www.bristol.ac.uk/alspac/researchers/our-data/. Genotyping of participants was reported by Warrington et al.^29^ Ethics approval for the study was obtained from the ALSPAC Ethics and Law Committee and the Local Research Ethics Committees (Refs: E1808/ E4168/ E5215/ E5691/ E5806/ 06/Q2006/53). Informed consent for the use of data collected via questionnaires and clinics was obtained from participants following the recommendations of the ALSPAC Ethics and Law Committee at the time. Consent for biological samples was collected in accordance with the Human Tissue Act (2004). The Generation R study^30^^,^^31^ is a population-based prospective cohort study from fetal life to adulthood. A total of 9778 pregnant women residing in Rotterdam, The Netherlands, with a delivery date from April 2002 until January 2006 were recruited. Genotyping and quality control performed in the Generation R study were reported by Medina-Gomez C et al.^32^ Ethics approval was obtained from the Medical Ethical Committee of Erasmus Medical Center, University Medical Center Rotterdam (Ref: MEC 217.595/2002/20). All participants provided written informed consent for each phase of the study (fetal, preschool, childhood, and adolescence period). Children provided consent from the age of 12 years onward, in accordance with Dutch Law. Figure 1 provides an overview of how each cohort contributed to the design of the study.

### GWAS Summary Statistics for Time Spent Outdoors

The UK Biobank participants were asked to report, “In a typical DAY in summer, how many hours do you spend outdoors?” (data field #1050). A GWAS for this measure of time spent outdoors, with the units “hours per day,” was performed in a sample of 280,891 participants of European ancestry. The GWAS participants were selected to ensure they were unrelated to the UK Biobank participants included in the Within Family GWAS Consortium GWAS of EduYears or the GWAS for spherical equivalent refractive error (see below). Details of the sample selection scheme and GWAS analysis are provided in Supplementary Note S1.

### GWAS Summary Statistics for Spherical Equivalent Refractive Error

Measurement of refractive error was introduced partway through the UK Biobank recruitment period: approximately a quarter of the UK Biobank participants had their refractive error assessed. Measurements were obtained with a Tomey RC5000 autorefractor (data fields #5084–5088). The average spherical equivalent of the 3 to 10 repeat readings per eye, in units of diopters, was calculated. The spherical equivalent refractive error of a participant was calculated as the average value for their two eyes.^15^ A GWAS for spherical equivalent refractive error was performed in a sample of 72,576 participants of European ancestry who were unrelated to any other participant in the UK Biobank. Details of the sample selection scheme and GWAS analysis parameters are provided in Supplementary Note S2.

### GWAS Summary Statistics for an Early Age-of-Onset of Spectacle Wear

The UK Biobank participants were asked to report, “What age did you first start to wear glasses or contact lenses?” (data field # 2217). Participants were classified as having an early age-of-onset of spectacle wear (EAOSW) if they reported first wearing glasses or contact lenses at or before the age of 15 years old (EAOSW = 1). All other participants were classified as not having an EAOSW (EAOSW = 0), including participants who did not answer this question (for example, because they never wore glasses or contact lenses). A GWAS for the binary outcome EAOSW was performed in the same sample of 72,576 participants of European ancestry in whom the GWAS for spherical equivalent refractive error was performed. Details of the GWAS analysis are provided in Supplementary Note S3.

### GWAS Summary Statistics for Years Spent in Education

Summary statistics from a GWAS of years spent in full-time education (EduYears) performed in a sample of 128,777 adult siblings of European ancestry by the Within Family GWAS Consortium^33^ were obtained from the UK Medical Research Council Integrative Epidemiology Unit (IEU) online database maintained by the University of Bristol (dataset ieu-b-4836).^34^^,^^35^ The regression coefficients were converted from units of standard deviation to units of years of full-time education, using the conversion factor 1 standard deviation = 2.52 years of education, obtained for the UK Biobank participants of European ancestry.

### Selection of Instrumental Variables and Derivation of Polygenic Scores

GWAS summary statistics were filtered to remove variants absent from the ALSPAC or the UK Biobank genotyping datasets and then clumped using PLINK^36^ to provide a set of independently associated variants (clumping parameters: linkage disequilibrium, r^2^ < 0.01 and genomic distance > 1000 kb). For the main Mendelian randomization analysis, GWAS variants associated with the exposure at *P* < 5 × 10^−8^ were selected; for a sensitivity analysis, this criterion was relaxed to *P* < 1 × 10^−7^ or *P* < 1 × 10^−6^. A polygenic score for years spent in education was derived from the GWAS summary statistics using LDpred2,^37^ as described in Supplementary Note S4. A polygenic score for time spent outdoors was derived using BOLT-LMM,^38^ as also described in Supplementary Note S4.

### Association of Polygenic Scores With Children's Time Spent Outdoors and Time Spent Reading

Questionnaires about the lifestyle of children participating in the ALSPAC and Generation R studies were completed by the study children themselves and their parents at regular intervals across childhood. In the ALSPAC study, questionnaire items relating to the time children spent outdoors typically followed the format, “How much time, on average, on a typical school weekday (weekend day) does your child spend out of doors in summer(winter).” In the Generation R study, questionnaire items typically asked: “How much time does your child, on average, spend outside during a school weekday (weekend day).” For comparison, responses from the lifestyle questionnaires that related to the time children spent reading were also retrieved. Linear regression was used to estimate the association of the polygenic score for time outdoors (in units of standard deviation) with the time children spent outdoors (hours per day) or with time children spent reading (hours per day), adjusting for sex and the first 10 genetic ancestry principal components. For comparison, the linear regression analyses were repeated using the polygenic score for years spent in education (in units of standard deviation). Details of the conversion of questionnaire responses from an ordinal to a pseudo-continuous scale, as well as details of the statistical analysis, are provided in Supplementary Note S5.

### Mendelian Randomization Analysis

The causal association of each exposure (years spent in education or time spent outdoors) and each outcome (spherical equivalent refractive error or the risk of EAOSW) was assessed by inverse-variance weighted, univariable two-sample Mendelian randomization. This was followed by an inverse-variance weighted MVMR analysis. The MVMR analysis provided an estimate of the direct effect of years spent in education on the outcome after adjusting for the effects of time spent outdoors. A range of sensitivity analyses were carried out to determine the robustness of the inverse-variance weighted Mendelian randomization analysis to the assumption of no horizontal pleiotropy; consistent findings across these different Mendelian randomization methods provides evidence of robustness to the presence of horizontal pleiotropy. Technical details of the Mendelian randomization and MRMR analyses are provided in Supplementary Note S6. The number of SNPs used as instrumental variables (IVs) for each exposure in the Mendelian randomization analyses is reported in Tables 1 and 2.

**Table 1.** Univariable and Multivariable Mendelian Randomization Results for the Outcome Spherical Equivalent Refractive Error

		EduYears					Time Outdoors				
MR/MVMR^f^	Method^a^	IVs^b^	Effect^c^	SE	95% CI	*P* Value	IVs^d^	Effect^e^	SE	95% CI	*P* Value
Univariable	IVW	24	−0.273	0.072	(−0.413 to −0.132)	1.4e–04	26	0.504	0.126	(0.258 to 0.750)	6.0e–05
	EGGER	24	−0.250	0.262	(−0.764 to 0.264)	3.4e–01	26	1.654	0.712	(0.260 to 3.049)	2.0e–02
	MEDIAN	24	−0.287	0.069	(−0.422 to −0.152)	3.0e–05	26	0.536	0.125	(0.292 to 0.781)	1.7e–05
	MBE	24	−0.321	0.130	(−0.575 to −0.067)	1.3e–02	26	0.928	0.249	(0.441 to 1.416)	1.9e–04
	PRESSO	22	−0.255	0.053	(−0.358 to −0.151)	9.1e–05	25	0.559	0.117	(0.329 to 0.788)	7.4e–05
	ROBUST	24	−0.252	0.064	(−0.377 to −0.126)	8.5e–05	26	0.513	0.134	(0.251 to 0.776)	1.2e–04
Multivariable	IVW	24	−0.165	0.077	(−0.317 to −0.014)	3.7e–02	26	0.478	0.122	(0.240 to 0.716)	2.7e–04
	EGGER	24	−0.175	0.083	(−0.338 to −0.012)	3.6e–02	26	0.542	0.238	(0.075 to 1.009)	2.3e–02
	MEDIAN	24	−0.178	0.081	(−0.337 to −0.019)	2.8e–02	26	0.389	0.139	(0.115 to 0.662)	5.3e–03
	MBE	22	−0.182	0.061	(−0.308 to −0.062)	1.7e–03	20	0.401	0.086	(0.161 to 0.570)	1.5e–03
	PRESSO	22	−0.173	0.069	(−0.308 to −0.039)	1.5e–02	25	0.465	0.106	(0.256 to 0.673)	7.3e–05
	ROBUST	24	−0.168	0.068	(−0.302 to −0.034)	1.4e–02	26	0.485	0.141	(0.209 to 0.761)	5.7e–04

a Methods were abbreviated as follows: IVW, inverse variance weighted MR and MVMR; EGGER, MR-Egger and MVMR-Egger analysis; MEDIAN, weighted median MR and MVMR; MBE, mode-based estimate MR and MVMR; PRESSO, MR-PRESSO; ROBUST, robust MR and MVMR.

b Number of instrumental variables used in analysis with years spent in education as an exposure.

c Estimated causal effect in units of Diopters per additional year spent in education.

d Number of instrumental variables used in analysis with time outdoors as an exposure.

e Estimated causal effect in units of Diopters per additional hour/day outdoors.

f The multivariable Mendelian randomization analyses simultaneously evaluated the effect of EduYears and time outdoors.

**Table 2.** Univariable and Multivariable Mendelian Randomization Results for the Outcome EAOSW

		EduYears					Time Outdoors				
MR/MVMR^f^	Method^a^	IVs^b^	Effect^c^	SE	95% CI	*P* Value	IVs^d^	Effect^e^	SE	95% CI	*P* Value
Univariable	IVW	24	0.167	0.062	(0.045 to 0.288)	7.2e–03	26	−0.209	0.074	(−0.353 to −0.065)	4.5e–03
	EGGER	24	0.076	0.232	(−0.379 to 0.531)	7.4e–01	26	−0.743	0.425	(−1.577 to 0.091)	8.1e–02
	MEDIAN	24	0.187	0.067	(0.057 to 0.318)	4.9e–03	26	−0.267	0.094	(−0.452 to −0.082)	4.7e–03
	MBE	24	0.282	0.190	(−0.091 to 0.654)	1.4e–01	26	−0.340	0.223	(−0.777 to 0.097)	1.3e–01
	PRESSO	22	0.156	0.057	(0.045 to 0.268)	1.2e–02	26	−0.209	0.074	(−0.353 to −0.065)	4.5e–03
	ROBUST	24	0.161	0.070	(0.025 to 0.298)	2.1e–02	26	−0.189	0.082	(−0.350 to −0.029)	2.1e–02
Multivariable	IVW	24	0.105	0.058	(−0.010 to 0.219)	7.9e–02	26	−0.205	0.092	(−0.385 to −0.025)	3.0e–02
	EGGER	24	0.113	0.063	(−0.010 to 0.237)	7.1e–02	26	−0.265	0.179	(−0.617 to 0.086)	1.4e–01
	MEDIAN	24	0.127	0.072	(−0.014 to 0.268)	7.6e–02	26	−0.223	0.108	(−0.433 to −0.012)	3.8e–02
	MBE	22	0.118	0.054	(0.004 to 0.224)	2.1e–02	25	−0.217	0.084	(−0.362 to −0.052)	3.5e–03
	PRESSO	22	0.104	0.054	(−0.003 to 0.210)	6.3e–02	26	−0.199	0.084	(−0.363 to −0.035)	2.2e–02
	ROBUST	24	0.106	0.064	(−0.019 to 0.231)	9.7e–02	26	−0.201	0.082	(−0.362 to −0.040)	1.4e–02

a Methods were abbreviated as follows: IVW, inverse variance weighted MR and MVMR; EGGER, MR-Egger and MVMR-Egger analysis; MEDIAN, weighted median MR and MVMR; MBE, mode-based estimate MR and MVMR; PRESSO, MR-PRESSO; ROBUST, robust MR and MVMR.

b Number of instrumental variables used in analysis with years spent in education as an exposure.

c Estimated causal effect in units of log odds ratio per additional year spent in education.

d Number of instrumental variables used in analysis with time outdoors as an exposure.

e Estimated causal effect in units of log odds ratio per additional hour/day outdoors.

f The multivariable Mendelian randomization analyses simultaneously evaluated the effect of EduYears and time outdoors.

## Results

### Evidence That Genetic Predisposition to Time Spent Outdoors Partially Tracks From Childhood to Adulthood

**Figure 2.**
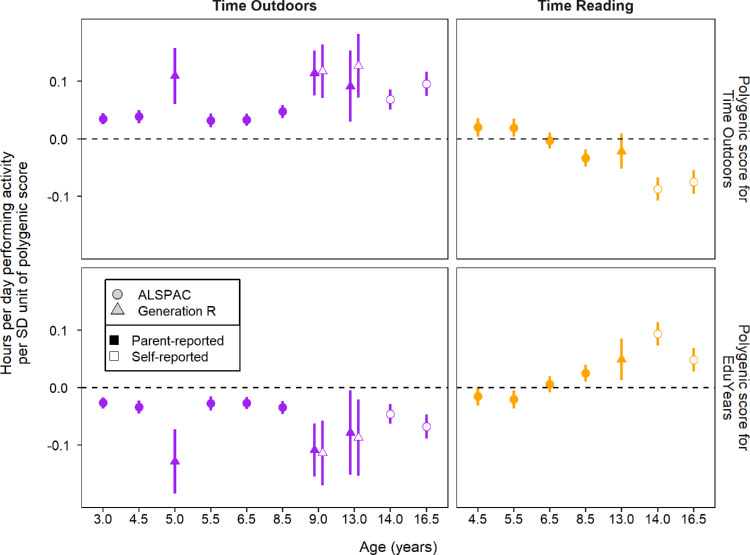
Association of polygenic scores for time outdoors or years spent in education with the time children spend outdoors or reading. Error bars show 95% CI. These results are presented in tabular form in Supplementary Tables S2 and S3.

A GWAS for time spent outdoors was performed using data for 9,572,557 genetic variants in a sample of 280,891 adult participants from the UK Biobank. Demographic characteristics of the sample are presented in Supplementary Table S1 and Figure S1. The GWAS analysis suggested time outdoors in adulthood had a genetic component: heritability estimate = 9.8% (standard error = 0.2%) for common genetic variants. To evaluate if an individual's genetic predisposition to time outdoors tracked from childhood to adulthood, a polygenic score for time outdoors was derived using the GWAS summary statistics for time outdoors. The polygenic score was tested in children from the ALSPAC and Generation R studies for an association with their self-reported or parent-reported time spent outdoors. In linear regression analyses, the polygenic score for time outdoors in adults was indeed positively associated with the time children spent outdoors (upper left panel of Fig. 2, Supplementary Tables S2, S3). The association persisted from the earliest age that information was ascertained (3 years) through until young adulthood, covering the period when myopia typically develops.^3^ The polygenic score for time outdoors was also associated with the time children spent reading books. However, beyond the age of 6.5 years, the association was negative, such that children with a higher genetic predisposition to spend time outdoors were found to spend less time reading. We also investigated if a polygenic score for years spent in education was associated with the time children spent outdoors and the time they spent reading. Interestingly, the polygenic score for years spent in education had a largely “mirror image” pattern of associations with the time children spent outdoors and reading (see lower panels of Fig. 2). Specifically, the polygenic score for years spent in education was associated with children spending less time outdoors and, beyond the age of 8.5 years, more time reading.

**Figure 3.**
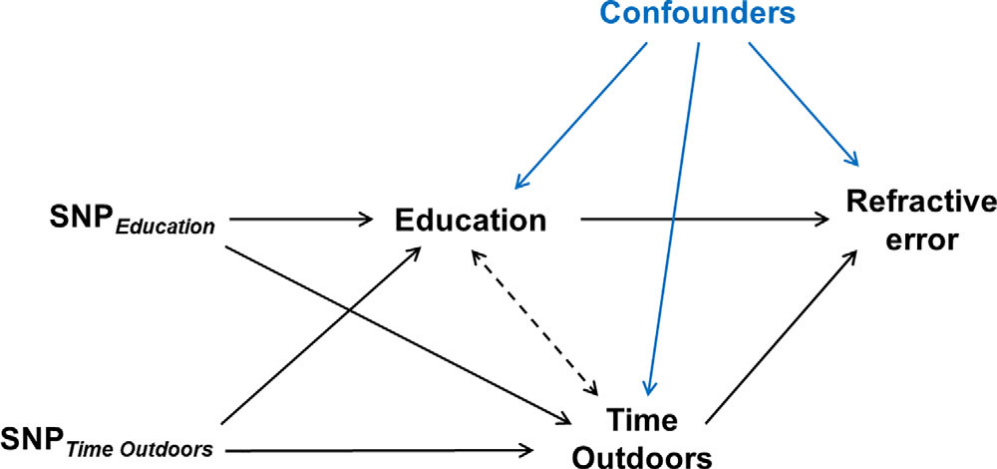
Principles of multivariable Mendelian randomization to estimate the causal effect of education on refractive error mediated by time spent outdoors. SNPs associated with years spent in education are used as instrumental variables to assess the causal effect of education on refractive error (*SNP_Education_* → *Education* → *Refractive error*). SNPs associated with time outdoors are used as instrumental variables to assess the causal effect of time outdoors on refractive error (*SNP_Time outdoors_* → *Time outdoors* → *Refractive error*). The causal pathway *Education* → *Time outdoors* → *Refractive error* is the pathway through which the causal effect of education on refractive error is mediated by time outdoors. The causal pathway *Time outdoors* → *Education* → *Refractive error* is the pathway through which the causal effect of time outdoors on refractive error is mediated by education. In a univariable Mendelian randomization analysis to assess the effect of education on refractive error, IVs for years spent in education acting through the pathway *SNP_Education_* → *Time outdoors* → *Refractive error* would yield a biased estimate. In a multivariable Mendelian randomization analysis, the use of IVs for both years spent in education and time outdoors would avoid this source of bias.

These findings suggested that GWAS variants associated with time outdoors in adulthood would serve as valid IVs for time spent outdoors during childhood, but only if the analysis took account of the additional association with years spent in education (see Fig. 3). Likewise, the use of IVs for years spent in education would be liable to produce biased causal effect estimates when used in isolation for Mendelian randomization studies, because of their association with time outdoors. Instead, our findings suggested that IVs for time spent outdoors and IVs for years spent in education should be used in a multivariable Mendelian randomization setting to take account of their potential association with both exposures (Fig. 3).

### Mendelian Randomization Analyses

**Figure 4.**
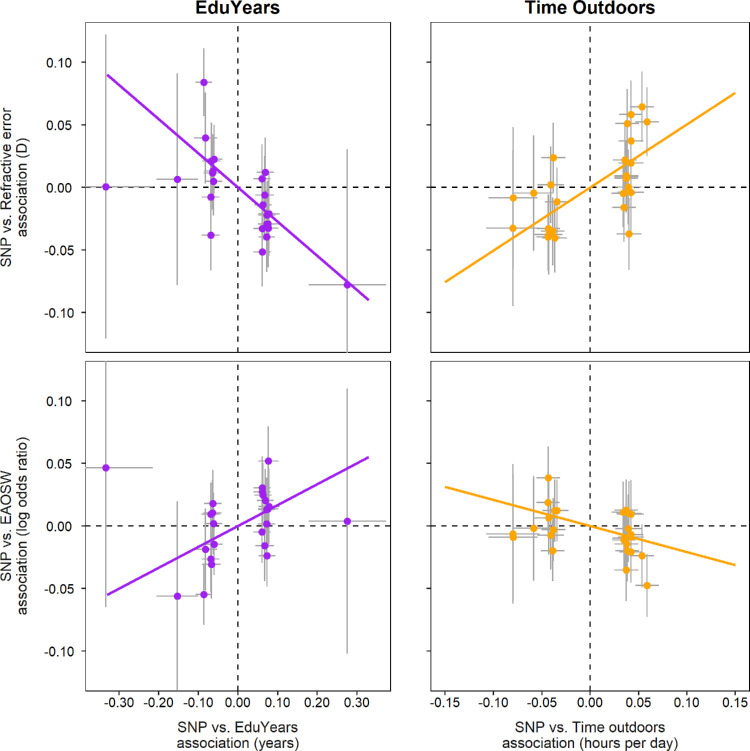
Univariable Mendelian randomization analysis of exposure to years spent in education (EduYears) or time spent outdoors on the outcomes of spherical equivalent refractive error or an early age-of-onset of spectacle wear (EAOSW). Each data point represents an independent genetic variant chosen for its strong association with either EduYears or time outdoors. Error bars show 95% CI. The slope of the solid line corresponds to the inverse-variance weighted Mendelian randomization (MR-IVW) causal effect estimate.

**Figure 5.**
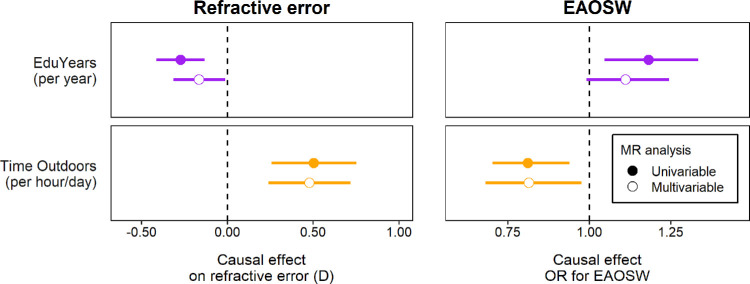
Comparison of univariable vs. multivariable Mendelian randomization analysis to estimate the causal effects of exposure to years spent in education (EduYears) and time spent outdoors on the outcomes of spherical equivalent refractive error, or an early age-of-onset of spectacle wear (EAOSW). Error bars show 95% CI.

The main univariable and multivariable Mendelian randomization analysis results are shown in Figures 4 and 5, and Tables 1 and 2. Full results are shown in Supplementary Tables S4 and S5. An inverse-variance weighted univariable Mendelian randomization analysis provided evidence that years spent in education had a causal association with spherical equivalent refractive error, consistent with two previous Mendelian randomization analyses of this relationship.^14^^,^^15^ An inverse-variance weighted univariable Mendelian randomization analysis also suggested that time outdoors had a causal association with spherical equivalent refractive error, consistent with robust evidence of a causal effect in RCTs.^11^^–^^13^ Inverse-variance weighted univariable analyses also provided evidence for a causal effect of years spent in education on the risk of EAOSW (as recently observed using data from the UK Biobank)^16^ and a causal effect of time outdoors on the risk of EAOSW.

However, as noted above, these univariable Mendelian randomization analyses may have produced biased results given that time outdoors may mediate some of the effects attributed to years spent in education, and vice versa. Therefore, a multivariable Mendelian randomization analysis was conducted to assess the evidence for such mediation. Comparison of an inverse-variance weighted multivariable versus univariable Mendelian randomization analysis suggested that time outdoors mediated approximately 40% of the estimated causal effect of years spent in education on spherical equivalent refractive error (−0.17 vs. −0.27 D for each additional year spent in education, for the multivariable versus univariable analysis, respectively; see Fig. 5). The findings were similar for the outcome EAOSW, with an estimated 39% of the estimated causal effect of years spent in education on the risk of an EAOSW acting through time outdoors (odds ratio [OR] = 1.11 vs. 1.18 for each additional year spent in education for the inverse-variance weighted multivariable versus univariable analysis; see Fig. 5). By contrast, we estimated that only 5% of the causal effect of time outdoors on spherical equivalent refractive error was mediated by years spent in education (+0.48 vs. +0.50 D for each additional hour per day outdoors, for the inverse-variance weighted multivariable versus univariable analysis) and there was minimal evidence for mediation by years spent in education in the estimated causal effect of time outdoors on the risk of EAOSW (OR = 0.81 vs. 0.81 for each additional hour per day outdoors for the inverse-variance weighted multivariable versus univariable Mendelian randomization analysis).

## Discussion

The major finding from this study was that the well-established causal effect of education on myopia was mediated in part by the influence of time spent outdoors. Indeed, 40% of the risk of myopia causally associated with years of education was mediated by spending less time outdoors. The confidence interval for the causal effect estimate of education was wide in the multivariable Mendelian randomization analysis (95% CI = −0.32 to −0.01 D per year of schooling), but did not include zero. Hence, these results suggest that spending more time indoors is an important risk factor for myopia but does not fully explain the causal association between years spent in education and refractive error (implying that other aspects of schooling, such as long periods of near viewing, may be important, too).

### Strengths and Limitations

Strengths of the study were: (1) the GWAS samples for both time outdoors and years spent in education were sufficiently large and well-powered to avoid a potential source of bias known as “weak instrument bias”; (2) independent samples of participants were analyzed in the GWAS for time outdoors, the GWAS for years spent in education, and the GWAS for spherical equivalent refractive error or EAOSW, which avoided potential bias from the use of overlapping samples of participants;^39^ (3) the IVs for years spent in education were obtained from a within-sibling GWAS analysis, which ensured these weightings represented genetic predisposition directly inherited by the participant. This is important in the context of years spent in education, because approximately 75% of the association between an individual's genotype and their educational attainment is mediated via an indirect route referred to as “genetic nurture” (whereby the genotype of parents or other family members influences their child's environment in such a way as to increase their years spent in education). By contrast, only 25% of the association between an individual's genotype and their educational attainment is mediated by direct, germline transmission of genetic variants,^40^ (4) as well as estimating the causal relationship of the exposures time outdoors and years spent in education on refractive error in adulthood, we also estimated their causal association with EAOSW. Because EAOSW “cases” were defined as individuals with an age-of-onset of spectacle wear below 16 years, this approach ensured that the effects of education and time outdoors were assessed during childhood, when myopia usually develops.^16^

To study the causal relationship between a specific exposure and an outcome, a valid IV must satisfy three rules.^41^ First, it must be strongly associated with the exposure; second, this association must be independent of any confounders of the exposure-outcome relationship; and third, the IV must only affect the outcome via its effect on the exposure. An important limitation of all Mendelian randomization studies is that the validity of the second and third rule is generally impossible to verify for GWAS variants. For example, the existence of “horizontal pleiotropy” – the independent effects of a genetic variant on multiple traits, which is a common feature of GWAS variants^42^ – would violate the third IV rule. Here, we performed sensitivity analyses using a range of Mendelian randomization methods that provide valid causal inferences even when some of the IVs have horizontally pleiotropic effects.^42^ In addition to reliance on the validity of the three fundamental IV assumptions, additional assumptions implicit in this work were: (i) SNPs identified in GWAS analyses of adults served as valid IVs for exposures in childhood, and (ii) the exposure versus outcome relationships of “time outdoors → refractive error” and “education → refractive error” were linear. We were able to validate that SNPs associated with time outdoors and educational attainment in adulthood were valid IVs for time spent outdoors and time spent reading, respectively, for children in recent birth cohorts (see Fig. 2); it was already established that SNPs associated with refractive error in adulthood are associated with refractive error development in childhood.^21^^–^^24^ Validation of the linearity of the exposure versus outcome relationships is an important topic for future work. Howe et al.^16^ first proposed EAOSW as an outcome to study the causal association of an exposure in childhood on the risk of myopia. These authors noted the paradox in studying the effects of EduYears in a sample that, prior to the age of 16 years, actually had no variation in years of schooling^16^; as an explanation, they suggested that genetic IVs for EduYears were not only capturing genetic predisposition to remain in education but also additional features of a genetic predisposition to education, which they described as “liability to educational attainment.” Hence, a further limitation of the current work is that it is unclear which specific aspects of liability to educational attainment were captured by SNPs associated with EduYears.

### Comparison With Previous Work

The “Tian-Tian Outdoor 120” education policy in Taiwan, which encouraged children to spend 2 hours outdoors each school day, coincided with a reversal of the increasing trend of myopia prevalence over previous years.^20^ Our findings support the interpretation that this change in myopia prevalence was causally related to children spending less time in the classroom and more time outdoors. However, whereas the Taiwan policy change study^20^ examined the effect of replacing 2 hours of indoor school teaching with 2 hours of outdoor activities, the current study examined the extent to which the causal association between years spent in education and myopia is mediated by spending time indoors in the classroom versus aspects of schooling, such as prolonged activity of near viewing.

A linear regression analysis of observational data from the UK Biobank reported^15^ that each year of education was associated with a −0.18 D shift in refractive error toward myopia, which is strikingly similar to the current causal effect estimate −0.17 D/year. Previous estimates of the causal effect of an additional year of schooling on refractive error obtained using univariable Mendelian randomisation^14^^,^^15^ have ranged from −0.27 to −0.92 D, while estimates ranging from −0.29 to −0.82 D have been reported using regression discontinuity.^17^^–^^19^ In general, estimates of the effect of education on myopia from children educated in China have been higher than those for children educated in Europe, consistent with the higher prevalence and severity of myopia observed in East and Southeast Asia compared to Europe.^14^^,^^15^^,^^17^^–^^19^ Causal effect estimates obtained using the regression discontinuity approach may also have been inflated by capturing additional environmental influences, such as the societal impact of education reforms that raised the school leaving age from 15 to 16 years in the United Kingdom,^17^ or the effects on children's behavior when they advance to a new school grade.^18^^,^^19^

## Conclusions

Genetic variants previously used in a wide variety of studies as IVs for years spent in education were found to be (inversely) associated with the time children spent outdoors, suggesting that previous Mendelian randomization studies examining the role of education in causing myopia would have included effects associated with spending time indoors. In support of this hypothesis, the causal effect on refractive error associated with an additional year of education was reduced by approximately 40%, from −0.27 D to −0.17 D, when account was taken of the effects of time outdoors. Education has well-established economic and public health benefits, therefore reducing the extent of children's education is not a credible strategy for curbing the increasing prevalence of myopia. Instead, our findings complement existing evidence suggesting that educating children outdoors, or re-designing school classrooms to introduce more daylight^43^ or to closely mimic the attributes of natural outdoor spaces^44^ has the potential to alleviate almost half of the adverse effects of education on myopia.

## Supplementary Material

Supplement 1
