# Supplementary material for: Time Spent Outdoors Partly Accounts for the Effect of Education on Myopia
Source: Invest Ophthalmol Vis Sci. 2023 Nov 27;64(14):38. doi: 10.1167/iovs.64.14.38 (PMC10683767; doi:10.1167/iovs.64.14.38)
Supplement: Supplement 1 [file iovs-64-14-38_s001.pdf]

Time spent outdoors partly accounts for the effect of education on myopia

Supplementary Information

Table of Contents

Note S1. GWAS for time spent outdoors in adults from UK Biobank 2

Note S2. GWAS for spherical equivalent refractive error in adults from UK Biobank 3

Note S3. GWAS for *EAOSW* in adults from UK Biobank 4

Note S4. Derivation of polygenic scores for *EduYears* and Time Outdoors 5

Note S5. ALSPAC/Generation R question-ire response coding and polygenic score a-lyses 6

Note S6. Technical details of Mendelian randomisation a-lyses 7

Note S7. Results of sensitivity a-lyses 8

Table S1. Demographic characteristics of UKB GWAS samples. 9

Table S2. Association of polygenic scores for time outdoors or EduYears with the time ALSPAC participants spent outdoors or reading. 10

Table S3. Association of polygenic scores for time outdoors or EduYears with the time Generation R participants spent outdoors or on near work 11

Table S4. Full MR and MVMR results for outcome: *Spherical equivalent refractive error*. 12

Table S5. Full MR and MVMR results for outcome: risk of *EAOSW*. 13

Table S6. Univariable MR testing for causal effect of refractive error on time outdoors. 14

Figure S1. Demographic characteristics of UKB GWAS samples. 15

Figure S2. Graph of SNP-Exposure vs. SNP-Outcome for GWAS variants with P<1e-07. 16

Figure S3. Graph of SNP-Exposure vs. SNP-Outcome for GWAS variants with P<1e-06. 17

Figure S4. Comparison of univariable vs. multivariable MR a-lysis for variants with P<5e-08. 18

Figure S5. Comparison of univariable vs. multivariable MR a-lysis for variants with P<1e-07. 19

Figure S6. Comparison of univariable vs. multivariable MR a-lysis for variants with P<1e-06. 20

UK Biobank Eye and Vision Consortium members 21

Supplementary References 23

Note S1. GWAS for time spent outdoors in adults from UK Biobank

Siblings who were both enrolled in UK Biobank were identified as participants whose genetic kinship [data field #22012] with any other participant was ≥ 1/(2^(5/2)) and ≤ 1/(2^(3/2)) and whose genetic identity-by-state zero (IBS0) sharing [data field #22013] was ≥ 0.0015 and ≤ 0.012, as described[45].

Participants were classified as having European ancestry if their first two genetic principal components (PCs) [data field #22009] were within the mean ± 10 standard deviations of all unrelated UK Biobank participants who self-reported their ethnicity as White British[46] and if they had genotype heterozygosity [data field #22004] also within the mean ± 10 standard deviations of all unrelated UK Biobank participants who self-reported their ethnicity as White British. We selected participants of European ancestry who had information available for time spent outdoors in summer [data field #1050], place-of-birth northing coordi-te [data field #129], place-of-birth easting coordi-te [data field #130] and Assessment Centre [data field #54] were filtered to exclude those related to a sibling and to exclude those who were included in the GWAS for spherical equivalent refractive error (see Note S2). This resulted in a sample of 280 891 individuals. Participants' age at the baseline assessment visit was calculated from their date of visit [data field #53] and their year of birth [data field #34] and month of birth [data field #52].

A GWAS for time outdoors in summer was performed with BOLT[47] for 9 572 557 imputed SNPs with minor allele frequency (MAF) ≥ 0.01, imputation quality metric (INFO) ≥ 0.8 and per variant genotyping call rate ≥ 0.95. Age, Age-squared, sex [data field #22001], northing coordi-te, easting coordi-te, genotyping array [data field #22000], the first 10 genetic ancestry PCs, and Assessment Centre (one-hot encoded) were included as covariates.

Note S2. GWAS for spherical equivalent refractive error in adults from UK Biobank

Calculation of participants' age at the baseline assessment visit and classification of individuals of European ancestry are described in Note S1. We selected unrelated participants [data field #22011] of European ancestry who had information available for non-cycloplegic autorefraction [data fields #5084–5088], place-of-birth northing coordi-te, place-of-birth easting coordi-te and Assessment Centre. This resulted in a sample of 72 576 individuals. The refractive error of each participant was taken as the *spherical equivalent refractive error* (sphere plus 0.5 × cylinder) averaged between the two eyes[48-50].

A GWAS for *spherical equivalent refractive error* was performed with BOLT[47] for 9 572 557 imputed SNPs with minor allele frequency (MAF) ≥ 0.01, imputation quality metric (INFO) ≥ 0.8 and per variant genotyping call rate ≥ 0.95. Age, Age-squared, sex, northing coordi-te, easting coordi-te, genotyping array, the first 10 genetic ancestry PCs, and Assessment Centre (one-hot encoded) were included as covariates.

Note S3. GWAS for *EAOSW* in adults from UK Biobank

The GWAS for EAOSW was performed in the same sample of 72 576 individuals at the GWAS for *spherical equivalent refractive error* (Note S2). Participants were classified as having an early age-of-onset of spectacle wear [data field # 2217] if they reported first wearing glasses or contact lenses at or before the age of 15 years old (*EAOSW* = 1). All other participants were classified as *not* having an early age-of-onset of spectacle wear (*EAOSW* = 0), including participants who did not answer this question (for example, because they never wore glasses or contact lenses).

A GWAS for the bi-ry outcome *EAOSW* was performed with the *glm* function in R. Age, Age-squared, sex, northing coordi-te, easting coordi-te, genotyping array, the first 10 genetic ancestry PCs, and Assessment Centre (one-hot encoded) were included as covariates.

Note S4. Derivation of polygenic scores for *EduYears* and Time Outdoors

(a) The polygenic score for time outdoors was derived by repeating the GWAS for time outdoors in summer (as described in Note S1) except that (i) only variants in the HapMap3 set (file 'map.rds' available at: https://doi.org/10.6084/m9.figshare.19213299) were included and (ii) the BOLT option --predBetasFile was selected to assume an infinitesimal model.

(b) The polygenic score for *EduYears* was derived using the summary statistics from the GWAS for *EduYears* reported by the Within Family GWAS Consortium[51] (dataset ieu-b-4836). The GWAS summary statistics were filtered to exclude variants not present in the HapMap3 set (file 'map.rds' above). Next, LDpred2 was used to account for linkage disequilibrium between variants, with default settings for an infinitesimal model (function *snp_ldpred2_inf* from the R package *bigsnpr*)[52].

Note S5. ALSPAC/Generation R question-ire response coding and polygenic score a-lyses

In the ALSPAC study, question-ire items relating to time spent outdoors followed the format, "How much time, on average, on a typical [school weekday / weekend day / school holiday] does your child spend out of doors in [summer / winter]." Items relating to time spent reading for pleasure followed the same format. The question-ire response options were: None; Less than one hour; One to two hours; Three or more hours. These responses were converted from an ordi-l to a pseudo-continuous scale by assigning values of 0, 0.5, 1.5 and 3 hours, respectively, to the above responses. The average time spent per day was calculated as: (hours on a weekday × 5/7) + (hours on a weekend day × 2/7). Assuming that summer comprised 13 weeks (6 weeks of holidays and 7 weeks non-holidays) and that winter comprised 13 weeks (3 weeks of holidays and 10 weeks non-holidays), the average time spent per day across the whole year was calculated as: (hours on a summer non-holiday × 7/26) + (hours on a summer holiday × 6/26) + (hours on a winter non-holiday × 10/26) + (hours on a winter holiday × 3/26).

In the Generation R study, question-ire items relating to time outdoors and near work typically had the following format: "How much time do you [does your child], on average, spend outside [reading] during a school weekday [weekend day]." The response options were: None, 0-30 minutes, 30-60 minutes, 1-2 hours, 3-4 hours, more than 4 hours. These responses were converted to a pseudo-continuous scale using the same approach as above.

A-lyses of polygenic score associations were carried out separately in the ALSPAC and Generation R cohorts. The polygenic score of each participant in the cohort was calculated using the --score function of PLINK[53] version 1.9, and was standardised to have a mean of zero and a standard deviation of one. The polygenic score for time outdoors was calculated using the beta coefficients described in Note S1(a), while the polygenic score for *EduYears* was calculated using the beta coefficients described in Note S1(b).

The following two regression equations were fitted for each of the time outdoors or time reading variables. All participants of European ancestry with genotype data and question-ire response data were included in the a-lysis:

$$Time\ in\ activity = PGS_{TimeOutdoors} + Sex + PC1 + PC2 \ldots + PC10 \qquad \text{(eq. 1)}$$

$$Time\ in\ activity = PGS_{EduYears} + Sex + PC1 + PC2 \ldots + PC10 \qquad \text{(eq. 2)}$$

where,

$PGS_{TimeOutdoors}$ is the standardized polygenic score for time outdoors, and $PGS_{EduYears}$ is the standardized polygenic score for $EduYears$.

Note S6. Technical details of Mendelian randomisation a-lyses

Steiger's test[54] was used to identify variants more strongly associated with the outcome than with the exposure; no variant failed the test (Steiger's test, $P > 0.05$). The correlation between IVs was computed using the command --*r* in PLINK[53] version 1.9. IVs with a squared correlation > 0.1 not already removed by the clumping procedure (due to being separated by more than 1000 kb or due to being closely-situated IVs for the two traits in an MVMR a-lysis) were pruned by removing the less strongly associated variant of each pair. MR a-lyses were carried out for the continuous outcome *spherical equivalent refractive error* or the bi-ry outcome *EAOSW*.

Univariable MR a-lysis methods included MR-IVW, Egger (MR-EGGER), weighted median (MR-MEDIAN) and mode-based (MR-MBE) a-lyses, which were performed using the *mr_ivw*, *mr_egger*, *mr_median* and *mr_mbe* functions, respectively, from the R package *MendelianRandomization* [55]. The MR-PRESSO a-lysis was performed using the *mr_presso* function from the R package *MRPRESSO* [56]. A robust (MR-ROBUST) a-lysis was performed with the *mvmr_robust* function from the R package robust-mvmr [57].

Multivariable MR a-lyses were carried out using the inverse variance weighting (MVMR-IVW), Egger (MVMRMR-EGGER) and weighted median (MVMR-MEDIAN) methods, performed with the *mr_mvivw*, *mr_mvegger* and *mvmr_median* functions, respectively, from the R package *MendelianRandomization* [55]. An MVMR mode-based a-lysis was performed with the *mv_mrmode* function from the R package *MVMRmode* [58]. An MVMR-PRESSO a-lysis was performed after removing the outlier IVs detected in the two univariable MR-PRESSO a-lyses[56]. A robust (MVMR-ROBUST) a-lysis was performed with the *mvmr_robust* function from the R package robust-mvmr [57].

Note S7. Results of sensitivity a-lyses

The results from the sensitivity a-lyses are shown in Supplementary Tables S4 and S5 and Supplementary Figures S2-S6. An F-statistic above 10 is indicative of robustness against weak instrument bias in multivariable Mendelian randomisation a-lyses[59]. The F-statistics from the inverse-variance weighted multivariable a-lyses were F = 12.1 for the years spent in education IVs and F = 19.6 s for the time outdoors IVs, suggesting they were robust to weak instrument bias (in the a-lyses using *spherical equivalent refractive error* as the outcome and in the a-lyses using the risk of *EOASW* as the outcome). There was strong evidence in the univariable and multivariable a-lyses of heterogeneity in SNP-exposure vs. SNP-outcome effect sizes: Cochran's Q-statistic was Q = 114.6, *P* = 1.40e-07, for the inverse-variance weighted multivariable a-lysis of *spherical equivalent refractive error*, and Q = 83.5, *P* = 8.30e-04, for the equivalent a-lysis of *EAOSW*. Such heterogeneity can be indicative of horizontal pleiotropy. However, repeating the univariable and multivariable a-lyses using a series of Mendelian randomisation methods designed to provide valid causal effect estimates in the presence of horizontal pleiotropy yielded highly comparable results to those from the inverse-variance weighted method. The confidence intervals were wide for the MR-Egger and MBE-MR methods (Tables 1 and 2), yet the causal effect estimates from these methods overlapped those from the other a-lysis methods. There was no indication of directio-l horizontal pleiotropy: MVMR-Egger intercept = 0.002 D, *P* = 0.83, for the outcome *spherical equivalent refractive error*, and MVMR-Egger intercept = -0.005, P = 0.54, for the outcome *EOASW*. Increasing the number of IVs for the two exposures by relaxing the GWAS p-value threshold for selecting SNPs from *P* < 5e-08 to either *P* < 1e-07 or *P* < 1e-06 yielded consistent causal effect estimates. Previous work failed to find a significant effect of refractive error on years spent in education[60,61], and here, a univariable Mendelian randomisation a-lysis provided little evidence that refractive error influenced the time children spent outdoors (Supplementary Table S6).

Table S1. Demographic characteristics of UKB GWAS samples.

Values are median (interquartile range) unless otherwise specified. Note that refractive error was not measured in the majority of the GWAS for time outdoors sample, as autorefraction was only introduced towards the end of the UK Biobank recruitment period.

Characteristics	GWAS for time outdoors	GWAS for refractive error
No. of participants	280 891	72 576
Female (%)	52.9	52.4
University degree (%)	31.6	36.2
Age (years)	58.5 (13.17)	59.75 (12.33)
Refractive error (D)	---	+0.12 (2.38)
Time outdoors in summer (hours/day)	3.5 (3.0)	3.0 (3.0)
Townsend deprivation index	168.0 (14.0)	169.0 (14.0)
Age completed full-time education	17.0 (5.0)	18.0 (5.0)

Table S2. Association of polygenic scores for time outdoors or EduYears with the time ALSPAC participants spent outdoors or reading.

(These data are plotted in Figure 1).

Polygenic score	Outcome trait	Age (years)	Report	N	BETA	SE	95% LCI	95% UCI	*P*
EduYears	Time Outdoors	3.0	Parent-reported	6692	-0.026	0.005	-0.036	-0.017	8.3e-08
EduYears	Time Outdoors	4.5	Parent-reported	6421	-0.034	0.006	-0.045	-0.023	4.6e-09
EduYears	Time Outdoors	5.5	Parent-reported	6176	-0.027	0.006	-0.039	-0.015	8.0e-06
EduYears	Time Outdoors	6.5	Parent-reported	5835	-0.027	0.005	-0.037	-0.016	7.7e-07
EduYears	Time Outdoors	8.5	Parent-reported	5910	-0.035	0.006	-0.046	-0.023	2.8e-09
EduYears	Time Outdoors	14.0	Self-reported	3785	-0.046	0.009	-0.063	-0.028	2.8e-07
EduYears	Time Outdoors	16.5	Self-reported	3733	-0.068	0.011	-0.089	-0.047	2.5e-10
EduYears	Time Reading	4.5	Parent-reported	6451	-0.015	0.008	-0.031	0.001	6.9e-02
EduYears	Time Reading	5.5	Parent-reported	6207	-0.020	0.008	-0.036	-0.004	1.2e-02
EduYears	Time Reading	6.5	Parent-reported	5854	0.006	0.007	-0.008	0.020	4.1e-01
EduYears	Time Reading	8.5	Parent-reported	5946	0.025	0.008	0.010	0.040	8.6e-04
EduYears	Time Reading	14.0	Self-reported	4521	0.093	0.010	0.073	0.114	6.0e-19
EduYears	Time Reading	16.5	Self-reported	3760	0.048	0.011	0.028	0.069	4.5e-06
Time Outdoors	Time Outdoors	3.0	Parent-reported	6692	0.035	0.005	0.025	0.045	1.1e-12
Time Outdoors	Time Outdoors	4.5	Parent-reported	6421	0.039	0.006	0.027	0.050	1.5e-11
Time Outdoors	Time Outdoors	5.5	Parent-reported	6176	0.032	0.006	0.020	0.0-	1.4e-07
Time Outdoors	Time Outdoors	6.5	Parent-reported	5835	0.033	0.005	0.023	0.0-	4.6e-10
Time Outdoors	Time Outdoors	8.5	Parent-reported	5910	0.048	0.006	0.036	0.059	3.8e-16
Time Outdoors	Time Outdoors	14.0	Self-reported	3785	0.068	0.009	0.051	0.086	5.2e-15
Time Outdoors	Time Outdoors	16.5	Self-reported	3733	0.096	0.011	0.075	0.117	5.3e-19
Time Outdoors	Time Reading	4.5	Parent-reported	6451	0.020	0.008	0.004	0.036	1.4e-02
Time Outdoors	Time Reading	5.5	Parent-reported	6207	0.019	0.008	0.004	0.035	1.6e-02
Time Outdoors	Time Reading	6.5	Parent-reported	5854	-0.003	0.007	-0.017	0.011	6.8e-01
Time Outdoors	Time Reading	8.5	Parent-reported	5946	-0.033	0.008	-0.048	-0.019	9.6e-06
Time Outdoors	Time Reading	14.0	Self-reported	4521	-0.087	0.010	-0.108	-0.067	4.5e-17
Time Outdoors	Time Reading	16.5	Self-reported	3760	-0.075	0.011	-0.096	-0.054	1.2e-12

Table S3. Association of polygenic scores for time outdoors or EduYears with the time Generation R participants spent outdoors or on near work.

(Some of these data are plotted in Figure 1).

**Polygenic score**	**Outcome trait**	**Age (years)**	**Report**	**N**	**BETA**	**SE**	**95% LCI**	**95% UCI**	***P***
EduYears	Time Outdoors	5	Parent-reported	2353	-0.129	0.029	-0.185	-0.072	7.58E-06
EduYears	Time Outdoors	9	Self-reported	1999	-0.114	0.029	-0.170	-0.058	7.12E-05
EduYears	Time Outdoors	9	Parent-reported	2201	-0.109	0.024	-0.155	-0.062	4.43E-06
EduYears	Time Outdoors	13	Parent-reported	863	-0.078	0.038	-0.152	-0.005	3.67E-02
EduYears	Time Outdoors	13	Self-reported	1760	-0.087	0.034	-0.154	-0.021	1.04E-02
EduYears	Time on Gaming	5	Parent-reported	2521	-0.026	0.007	-0.040	-0.011	5.89E-04
EduYears	Time watching TV	5	Parent-reported	2525	-0.105	0.017	-0.139	-0.071	1.36E-09
EduYears	Time on Gaming	9	Parent-reported	2156	-0.020	0.017	-0.053	0.013	2.36E-01
EduYears	Time watching TV	9	Parent-reported	2174	-0.137	0.024	-0.184	-0.089	1.92E-08
EduYears	Home Work	13	Parent-reported	1791	0.003	0.037	-0.070	0.075	9.43E-01
EduYears	Near Work	13	Parent-reported	932	0.080	0.068	-0.053	0.213	2.40E-01
EduYears	Time on Tablet	13	Parent-reported	938	-0.016	0.059	-0.132	0.100	7.89E-01
EduYears	Time Reading	13	Parent-reported	994	0.049	0.019	0.013	0.085	8.53E-03
Time Outdoors	Time Outdoors	5	Parent-reported	2353	0.109	0.025	0.061	0.158	1.10E-05
Time Outdoors	Time Outdoors	9	Self-reported	1999	0.118	0.024	0.071	0.164	8.02E-07
Time Outdoors	Time Outdoors	9	Parent-reported	2201	0.114	0.020	0.076	0.153	8.92E-09
Time Outdoors	Time Outdoors	13	Parent-reported	863	0.091	0.032	0.030	0.153	3.86E-03
Time Outdoors	Time Outdoors	13	Self-reported	1760	0.127	0.028	0.072	0.182	7.-E-06
Time Outdoors	Time on Gaming	5	Parent-reported	2521	0.019	0.006	0.006	0.031	3.38E-03
Time Outdoors	Time watching TV	5	Parent-reported	2525	0.074	0.015	0.0-	0.103	9.15E-07
Time Outdoors	Time on Gaming	9	Parent-reported	2156	0.033	0.014	0.006	0.061	1.81E-02
Time Outdoors	Time watching TV	9	Parent-reported	2174	0.093	0.020	0.053	0.133	4.97E-06
Time Outdoors	Home Work	13	Parent-reported	1791	0.028	0.031	-0.032	0.089	3.59E-01
Time Outdoors	Near Work	13	Parent-reported	932	-0.002	0.057	-0.113	0.110	9.77E-01
Time Outdoors	Time on Tablet	13	Parent-reported	938	0.097	0.049	0.000	0.194	5.06E-02
Time Outdoors	Time Reading	13	Parent-reported	994	-0.021	0.016	-0.052	0.009	1.69E-01

Table S4. Full MR and MVMR results for outcome: *Spherical equivalent refractive error*.

Results are expressed in units of Diopters.

IV Pval threshold	MR/ MVMR	Method	Time Outdoors (per hour per day)											Years spent in education (years)										
			IVs	Effect	SE	95% CI	*P*	F-stat	Q-stat	Q-pval	Egger Intercept	Egger P	PRESSO outliers	IVs	Effect	SE	95% CI	*P*	F-stat	Q-stat	Q-pval	Egger Intercept	Egger P	PRESSO outliers
1.0e-06	MR	IVW	72	0.378	0.069	(0.244 to 0.512)	3.5e-08	-	146.3	3.8e-07	-	-	-	70	-0.175	0.046	(-0.265 to -0.086)	1.2e-04	-	181.1	5.3e-12	-	-	-
1.0e-06	MR	EGGER	72	0.515	0.282	(-0.038 to 1.068)	6.8e-02	-	145.7	3.0e-07	-0.006	0.62	-	70	-0.082	0.157	(-0.390 to 0.226)	6.0e-01	-	180.1	4.5e-12	-0.007	0.53	-
1.0e-06	MR	MEDIAN	72	0.348	0.078	(0.195 to 0.501)	8.3e-06	-	-	-	-	-	-	70	-0.135	0.046	(-0.225 to -0.045)	3.3e-03	-	-	-	-	-	-
1.0e-06	MR	MBE	72	0.260	0.287	(-0.303 to 0.823)	3.7e-01	-	-	-	-	-	-	70	-0.035	0.121	(-0.272 to 0.202)	7.7e-01	-	-	-	-	-	-
1.0e-06	MR	PRESSO	72	0.378	0.069	(0.244 to 0.512)	3.5e-08	-	-	-	-	-	0	68	-0.163	0.042	(-0.246 to -0.080)	2.6e-04	-	-	-	-	-	2
1.0e-06	MR	ROBUST	72	0.358	0.084	(0.194 to 0.522)	1.8e-05	-	-	-	-	-	-	70	-0.152	0.048	(-0.245 to -0.059)	1.4e-03	-	-	-	-	-	-
1.0e-06	MVMR	IVW	72	0.384	0.073	(0.241 to 0.528)	5.7e-07	13.6	291.9	6.6e-13	-	-	-	70	-0.104	0.044	(-0.190 to -0.017)	2.0e-02	10.8	291.9	6.6e-13	-	-	-
1.0e-06	MVMR	EGGER	72	0.464	0.129	(0.211 to 0.718)	3.3e-04	-	301.2	5.2e-14	-0.003	0.45	-	70	-0.115	0.047	(-0.207 to -0.024)	1.4e-02	-	301.2	5.2e-14	-0.003	0.45	-
1.0e-06	MVMR	MEDIAN	72	0.298	0.087	(0.127 to 0.469)	6.4e-04	-	-	-	-	-	-	70	-0.119	0.052	(-0.221 to -0.017)	2.2e-02	-	-	-	-	-	-
1.0e-06	MVMR	MBE	71	0.405	0.074	(0.239 to 0.550)	9.5e-16	-	-	-	-	-	-	59	-0.085	0.037	(-0.150 to -0.012)	2.9e-02	-	-	-	-	-	-
1.0e-06	MVMR	PRESSO	72	0.355	0.071	(0.216 to 0.494)	1.7e-06	-	-	-	-	-	0	68	-0.104	0.043	(-0.189 to -0.019)	1.8e-02	-	-	-	-	-	2
1.0e-06	MVMR	ROBUST	72	0.368	0.082	(0.208 to 0.528)	6.4e-06	-	-	-	-	-	-	70	-0.094	0.045	(-0.182 to -0.006)	3.6e-02	-	-	-	-	-	-
1.0e-07	MR	IVW	30	0.443	0.115	(0.217 to 0.670)	1.2e-04	-	88.0	7.4e-08	-	-	-	29	-0.292	0.069	(-0.427 to -0.157)	2.4e-05	-	80.1	6.5e-07	-	-	-
1.0e-07	MR	EGGER	30	0.877	0.613	(-0.324 to 2.078)	1.5e-01	-	86.4	7.2e-08	-0.018	0.47	-	29	-0.234	0.262	(-0.748 to 0.280)	3.7e-01	-	79.9	3.9e-07	-0.004	0.82	-
1.0e-07	MR	MEDIAN	30	0.360	0.119	(0.126 to 0.593)	2.6e-03	-	-	-	-	-	-	29	-0.279	0.066	(-0.409 to -0.150)	2.5e-05	-	-	-	-	-	-
1.0e-07	MR	MBE	30	0.119	0.349	(-0.564 to 0.802)	7.3e-01	-	-	-	-	-	-	29	-0.302	0.140	(-0.576 to -0.028)	3.1e-02	-	-	-	-	-	-
1.0e-07	MR	PRESSO	29	0.490	0.109	(0.276 to 0.704)	1.1e-04	-	-	-	-	-	1	27	-0.279	0.057	(-0.391 to -0.167)	4.6e-05	-	-	-	-	-	2
1.0e-07	MR	ROBUST	30	0.441	0.133	(0.181 to 0.701)	8.9e-04	-	-	-	-	-	-	29	-0.275	0.071	(-0.413 to -0.137)	9.7e-05	-	-	-	-	-	-
1.0e-07	MVMR	IVW	30	0.381	0.118	(0.150 to 0.611)	2.0e-03	17.3	144.5	9.3e-10	-	-	-	29	-0.211	0.072	(-0.352 to -0.069)	5.0e-03	11.9	144.5	9.3e-10	-	-	-
1.0e-07	MVMR	EGGER	30	0.477	0.208	(0.069 to 0.886)	2.2e-02	-	151.8	9.0e-11	-0.004	0.57	-	29	-0.224	0.076	(-0.373 to -0.074)	3.3e-03	-	151.8	9.0e-11	-0.004	0.57	-
1.0e-07	MVMR	MEDIAN	30	0.253	0.132	(-0.005 to 0.512)	5.5e-02	-	-	-	-	-	-	29	-0.234	0.075	(-0.381 to -0.088)	1.7e-03	-	-	-	-	-	-
1.0e-07	MVMR	MBE	25	0.300	0.148	(0.100 to 0.590)	2.9e-03	-	-	-	-	-	-	26	-0.196	0.057	(-0.325 to -0.085)	1.1e-04	-	-	-	-	-	-
1.0e-07	MVMR	PRESSO	29	0.364	0.107	(0.154 to 0.573)	1.2e-03	-	-	-	-	-	1	27	-0.220	0.066	(-0.350 to -0.090)	1.6e-03	-	-	-	-	-	2
1.0e-07	MVMR	ROBUST	30	0.372	0.147	(0.084 to 0.660)	1.1e-02	-	-	-	-	-	-	29	-0.211	0.071	(-0.349 to -0.073)	2.8e-03	-	-	-	-	-	-
5.0e-08	MR	IVW	26	0.504	0.126	(0.258 to 0.750)	6.0e-05	-	80.2	1.1e-07	-	-	-	24	-0.273	0.072	(-0.413 to -0.132)	1.4e-04	-	61.7	2.1e-05	-	-	-
5.0e-08	MR	EGGER	26	1.654	0.712	(0.260 to 3.049)	2.0e-02	-	72.1	1.1e-06	-0.048	0.10	-	24	-0.250	0.262	(-0.764 to 0.264)	3.4e-01	-	61.7	1.2e-05	-0.002	0.93	-
5.0e-08	MR	MEDIAN	26	0.536	0.125	(0.292 to 0.781)	1.7e-05	-	-	-	-	-	-	24	-0.287	0.069	(-0.422 to -0.152)	3.0e-05	-	-	-	-	-	-
5.0e-08	MR	MBE	26	0.928	0.249	(0.441 to 1.416)	1.9e-04	-	-	-	-	-	-	24	-0.321	0.130	(-0.575 to -0.067)	1.3e-02	-	-	-	-	-	-
5.0e-08	MR	PRESSO	25	0.559	0.117	(0.329 to 0.788)	7.4e-05	-	-	-	-	-	1	22	-0.255	0.053	(-0.358 to -0.151)	9.1e-05	-	-	-	-	-	2
5.0e-08	MR	ROBUST	26	0.513	0.134	(0.251 to 0.776)	1.2e-04	-	-	-	-	-	-	24	-0.252	0.064	(-0.377 to -0.126)	8.5e-05	-	-	-	-	-	-
5.0e-08	MVMR	IVW	26	0.478	0.122	(0.240 to 0.716)	2.7e-04	19.6	114.6	1.4e-07	-	-	-	24	-0.165	0.077	(-0.317 to -0.014)	3.7e-02	12.1	114.6	1.4e-07	-	-	-
5.0e-08	MVMR	EGGER	26	0.542	0.238	(0.075 to 1.009)	2.3e-02	-	121.5	1.6e-08	-0.003	0.75	-	24	-0.175	0.083	(-0.338 to -0.012)	3.6e-02	-	121.5	1.6e-08	-0.003	0.75	-
5.0e-08	MVMR	MEDIAN	26	0.389	0.139	(0.115 to 0.662)	5.3e-03	-	-	-	-	-	-	24	-0.178	0.081	(-0.337 to -0.019)	2.8e-02	-	-	-	-	-	-
5.0e-08	MVMR	MBE	20	0.401	0.086	(0.161 to 0.570)	1.5e-03	-	-	-	-	-	-	22	-0.182	0.061	(-0.308 to -0.062)	1.7e-03	-	-	-	-	-	-
5.0e-08	MVMR	PRESSO	25	0.465	0.106	(0.256 to 0.673)	7.3e-05	-	-	-	-	-	1	22	-0.173	0.069	(-0.308 to -0.039)	1.5e-02	-	-	-	-	-	2
5.0e-08	MVMR	ROBUST	26	0.485	0.141	(0.209 to 0.761)	5.7e-04	-	-	-	-	-	-	24	-0.168	0.068	(-0.302 to -0.034)	1.4e-02	-	-	-	-	-	-

Abbreviations: IVW = Inverse variance weighted MR and MVMR; EGGER = MR-Egger and MVMR-Egger a-lysis; MEDIAN = weighted median MR and MVMR; MBE = mode-based estimate MR and MVMR; PRESSO = MR-PRESSO; ROBUST = robust MR and MVMR.

Table S5. Full MR and MVMR results for outcome: risk of *EAOSW*.

Results are expressed in units of log odds ratio.

			Time Outdoors (per hour per day)											Years spent in education (years)										
**IV Pval threshold**	**MR/ MVMR**	**Method**	**IVs**	**Effect**	**SE**	**95% CI**	***P***	**F-stat**	**Q-stat**	**Q-pval**	**Egger Intercept**	**Egger P**	**PRESSO outliers**	**IVs**	**Effect**	**SE**	**95% CI**	***P***	**F-stat**	**Q-stat**	**Q-pval**	**Egger Intercept**	**Egger P**	**PRESSO outliers**
1.0e-06	MR	IVW	72	-0.292	0.051	(-0.393 to -0.191)	1.3e-08	-	100.4	1.2e-02	-	-	-	70	0.123	0.031	(0.063 to 0.183)	6.4e-05	-	99.7	9.2e-03	-	-	-
1.0e-06	MR	EGGER	72	-0.507	0.213	(-0.926 to -0.089)	1.7e-02	-	98.8	1.3e-02	0.009	0.30	-	70	0.050	0.107	(-0.161 to 0.260)	6.4e-01	-	99.0	8.4e-03	0.005	0.48	-
1.0e-06	MR	MEDIAN	72	-0.308	0.066	(-0.438 to -0.178)	3.3e-06	-	-	-	-	-	-	70	0.113	0.039	(0.036 to 0.189)	3.8e-03	-	-	-	-	-	-
1.0e-06	MR	MBE	72	-0.368	0.192	(-0.7- to 0.008)	5.5e-02	-	-	-	-	-	-	70	0.008	0.112	(-0.212 to 0.227)	9.5e-01	-	-	-	-	-	-
1.0e-06	MR	PRESSO	72	-0.292	0.051	(-0.393 to -0.191)	1.3e-08	-	-	-	-	-	0	69	0.106	0.029	(0.049 to 0.164)	5.5e-04	-	-	-	-	-	1
1.0e-06	MR	ROBUST	72	-0.283	0.055	(-0.390 to -0.175)	2.7e-07	-	-	-	-	-	-	70	0.109	0.034	(0.043 to 0.176)	1.2e-03	-	-	-	-	-	-
1.0e-06	MVMR	IVW	72	-0.272	0.051	(-0.373 to -0.171)	4.8e-07	13.6	177.7	1.5e-02	-	-	-	70	0.090	0.031	(0.029 to 0.151)	4.2e-03	10.8	177.7	1.5e-02	-	-	-
1.0e-06	MVMR	EGGER	72	-0.378	0.090	(-0.555 to -0.201)	2.9e-05	-	179.4	1.2e-02	0.004	0.15	-	70	0.106	0.033	(0.041 to 0.170)	1.3e-03	-	179.4	1.2e-02	0.004	0.15	-
1.0e-06	MVMR	MEDIAN	72	-0.286	0.070	(-0.423 to -0.149)	4.5e-05	-	-	-	-	-	-	70	0.085	0.042	(0.003 to 0.167)	4.2e-02	-	-	-	-	-	-
1.0e-06	MVMR	MBE	71	-0.258	0.046	(-0.358 to -0.167)	1.0e-08	-	-	-	-	-	-	67	0.074	0.028	(0.016 to 0.129)	1.6e-02	-	-	-	-	-	-
1.0e-06	MVMR	PRESSO	72	-0.262	0.051	(-0.362 to -0.163)	7.9e-07	-	-	-	-	-	0	69	0.082	0.031	(0.022 to 0.142)	8.4e-03	-	-	-	-	-	1
1.0e-06	MVMR	ROBUST	72	-0.272	0.054	(-0.378 to -0.166)	5.1e-07	-	-	-	-	-	-	70	0.086	0.031	(0.025 to 0.147)	5.4e-03	-	-	-	-	-	-
1.0e-07	MR	IVW	30	-0.200	0.067	(-0.332 to -0.068)	3.0e-03	-	36.9	1.5e-01	-	-	-	29	0.188	0.051	(0.087 to 0.289)	2.5e-04	-	54.2	2.1e-03	-	-	-
1.0e-07	MR	EGGER	30	-0.732	0.346	(-1.410 to -0.054)	3.4e-02	-	33.9	2.0e-01	0.023	0.12	-	29	0.113	0.200	(-0.280 to 0.506)	5.7e-01	-	53.9	1.6e-03	0.005	0.70	-
1.0e-07	MR	MEDIAN	30	-0.216	0.090	(-0.393 to -0.040)	1.6e-02	-	-	-	-	-	-	29	0.185	0.060	(0.068 to 0.302)	2.0e-03	-	-	-	-	-	-
1.0e-07	MR	MBE	30	-0.333	0.220	(-0.765 to 0.099)	1.3e-01	-	-	-	-	-	-	29	0.238	0.166	(-0.088 to 0.564)	1.5e-01	-	-	-	-	-	-
1.0e-07	MR	PRESSO	30	-0.200	0.067	(-0.332 to -0.068)	3.0e-03	-	-	-	-	-	0	28	0.157	0.049	(0.061 to 0.253)	3.4e-03	-	-	-	-	-	1
1.0e-07	MR	ROBUST	30	-0.179	0.078	(-0.331 to -0.026)	2.2e-02	-	-	-	-	-	-	29	0.180	0.057	(0.068 to 0.293)	1.7e-03	-	-	-	-	-	-
1.0e-07	MVMR	IVW	30	-0.202	0.080	(-0.359 to -0.045)	1.4e-02	17.3	84.8	7.9e-03	-	-	-	29	0.121	0.049	(0.025 to 0.218)	1.7e-02	11.9	84.8	7.9e-03	-	-	-
1.0e-07	MVMR	EGGER	30	-0.314	0.141	(-0.590 to -0.038)	2.6e-02	-	85.1	7.3e-03	0.005	0.33	-	29	0.136	0.052	(0.035 to 0.238)	8.3e-03	-	85.1	7.3e-03	0.005	0.33	-
1.0e-07	MVMR	MEDIAN	30	-0.176	0.106	(-0.384 to 0.032)	9.7e-02	-	-	-	-	-	-	29	0.146	0.067	(0.015 to 0.277)	2.9e-02	-	-	-	-	-	-
1.0e-07	MVMR	MBE	29	-0.190	0.088	(-0.320 to -0.017)	2.6e-02	-	-	-	-	-	-	29	0.132	0.045	(0.025 to 0.220)	2.6e-03	-	-	-	-	-	-
1.0e-07	MVMR	PRESSO	30	-0.185	0.077	(-0.336 to -0.034)	2.0e-02	-	-	-	-	-	0	28	0.104	0.048	(0.011 to 0.198)	3.3e-02	-	-	-	-	-	1
1.0e-07	MVMR	ROBUST	30	-0.194	0.077	(-0.345 to -0.042)	1.2e-02	-	-	-	-	-	-	29	0.118	0.052	(0.016 to 0.220)	2.4e-02	-	-	-	-	-	-
5.0e-08	MR	IVW	26	-0.209	0.074	(-0.353 to -0.065)	4.5e-03	-	33.8	1.1e-01	-	-	-	24	0.167	0.062	(0.045 to 0.288)	7.2e-03	-	56.1	1.4e-04	-	-	-
5.0e-08	MR	EGGER	26	-0.743	0.425	(-1.577 to 0.091)	8.1e-02	-	31.7	1.4e-01	0.022	0.20	-	24	0.076	0.232	(-0.379 to 0.531)	7.4e-01	-	55.7	9.6e-05	0.007	0.69	-
5.0e-08	MR	MEDIAN	26	-0.267	0.094	(-0.452 to -0.082)	4.7e-03	-	-	-	-	-	-	24	0.187	0.067	(0.057 to 0.318)	4.9e-03	-	-	-	-	-	-
5.0e-08	MR	MBE	26	-0.340	0.223	(-0.777 to 0.097)	1.3e-01	-	-	-	-	-	-	24	0.282	0.190	(-0.091 to 0.654)	1.4e-01	-	-	-	-	-	-
5.0e-08	MR	PRESSO	26	-0.209	0.074	(-0.353 to -0.065)	4.5e-03	-	-	-	-	-	0	22	0.156	0.057	(0.045 to 0.268)	1.2e-02	-	-	-	-	-	2
5.0e-08	MR	ROBUST	26	-0.189	0.082	(-0.350 to -0.029)	2.1e-02	-	-	-	-	-	-	24	0.161	0.070	(0.025 to 0.298)	2.1e-02	-	-	-	-	-	-
5.0e-08	MVMR	IVW	26	-0.205	0.092	(-0.385 to -0.025)	3.0e-02	19.6	83.5	8.3e-04	-	-	-	24	0.105	0.058	(-0.010 to 0.219)	7.9e-02	12.1	83.5	8.3e-04	-	-	-
5.0e-08	MVMR	EGGER	26	-0.265	0.179	(-0.617 to 0.086)	1.4e-01	-	84.8	6.1e-04	0.003	0.69	-	24	0.113	0.063	(-0.010 to 0.237)	7.1e-02	-	84.8	6.1e-04	0.003	0.69	-
5.0e-08	MVMR	MEDIAN	26	-0.223	0.108	(-0.433 to -0.012)	3.8e-02	-	-	-	-	-	-	24	0.127	0.072	(-0.014 to 0.268)	7.6e-02	-	-	-	-	-	-
5.0e-08	MVMR	MBE	25	-0.217	0.084	(-0.362 to -0.052)	3.5e-03	-	-	-	-	-	-	22	0.118	0.054	(0.004 to 0.224)	2.1e-02	-	-	-	-	-	-
5.0e-08	MVMR	PRESSO	26	-0.199	0.084	(-0.363 to -0.035)	2.2e-02	-	-	-	-	-	0	22	0.104	0.054	(-0.003 to 0.210)	6.3e-02	-	-	-	-	-	2
5.0e-08	MVMR	ROBUST	26	-0.201	0.082	(-0.362 to -0.040)	1.4e-02	-	-	-	-	-	-	24	0.106	0.064	(-0.019 to 0.231)	9.7e-02	-	-	-	-	-	-

Abbreviations: IVW = Inverse variance weighted MR and MVMR; EGGER = MR-Egger and MVMR-Egger a-lysis; MEDIAN = weighted median MR and MVMR; MBE = mode-based estimate MR and MVMR; PRESSO = MR-PRESSO; ROBUST = robust MR and MVMR.

Table S6. Univariable MR testing for causal effect of refractive error on time outdoors.

(Results are expressed in units of hours per day outdoors per Diopter of refractive error).

Method	Exposure	Outcome	IVs	BETA	SE	95% CI	*P*
MR-IVW	Refractive Error	Time Outdoors	111	0.008	0.008	(-0.008 to 0.023)	0.327
MR-EGGER	Refractive Error	Time Outdoors	111	-0.036	0.021	(-0.077 to 0.005)	0.087
MR-MEDIAN	Refractive Error	Time Outdoors	111	0.000	0.009	(-0.018 to 0.017)	0.958
MR-MBE	Refractive Error	Time Outdoors	111	-0.002	0.017	(-0.034 to 0.031)	0.922
MR-PRESSO	Refractive Error	Time Outdoors	110	0.004	0.007	(-0.009 to 0.018)	0.508
MR-ROBUST	Refractive Error	Time Outdoors	111	0.003	0.007	(-0.010 to 0.017)	0.655

Abbreviations: MR-IVW = Inverse variance weighted MR; MR-MEDIAN = weighted median MR; MR-MBE = mode-based estimate MR.

Figure S1. Demographic characteristics of UKB GWAS samples.

These data are presented in Table S1. Note that refractive error was not measured in the majority of the GWAS for time outdoors sample, as autorefraction was only introduced towards the end of the UK Biobank recruitment period.

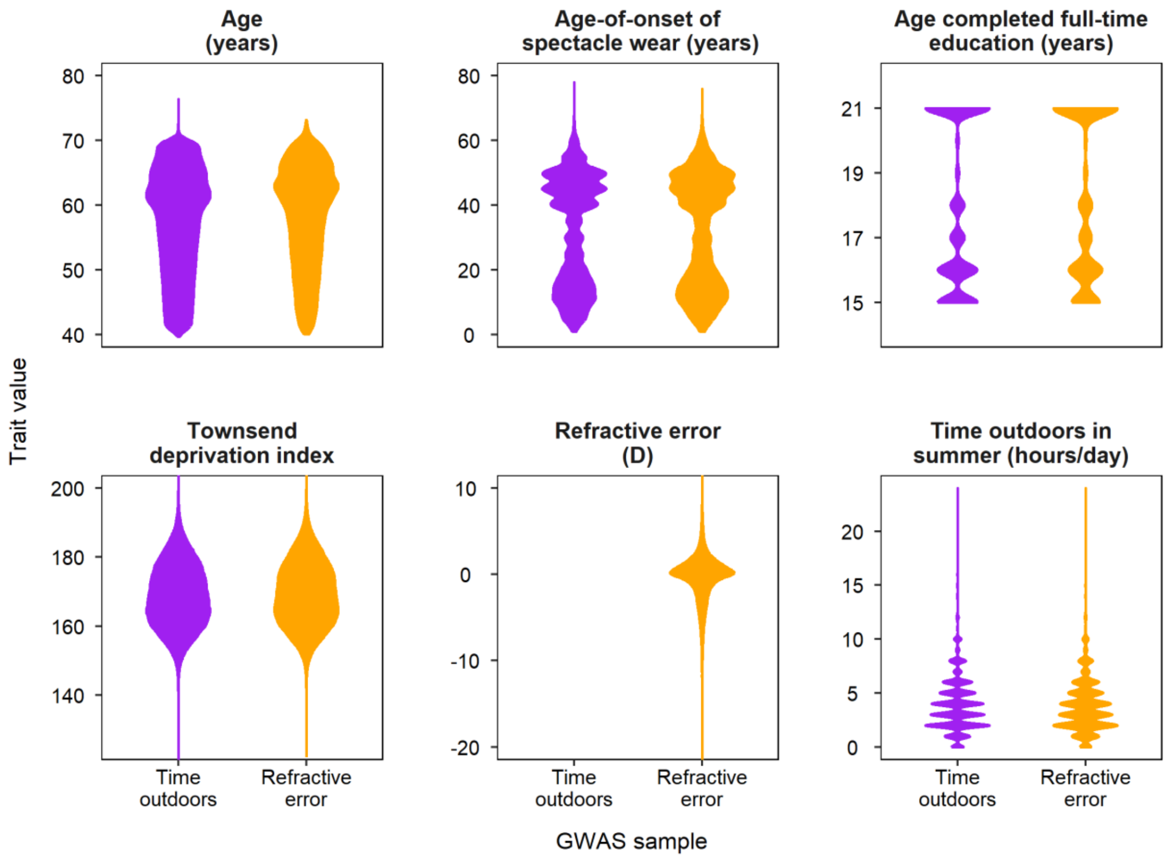

Figure S2. Graph of SNP-Exposure vs. SNP-Outcome for GWAS variants with P<1e-07. Univariable Mendelian randomisation a-lysis of the exposure EduYears or time spent outdoors on the outcome of either spherical equivalent refractive error or an EAOSW. Error bars show 95% CI. The slope of the solid line corresponds to the inverse-variance weighted Mendelian randomisation (MR-IVW) causal effect estimate.

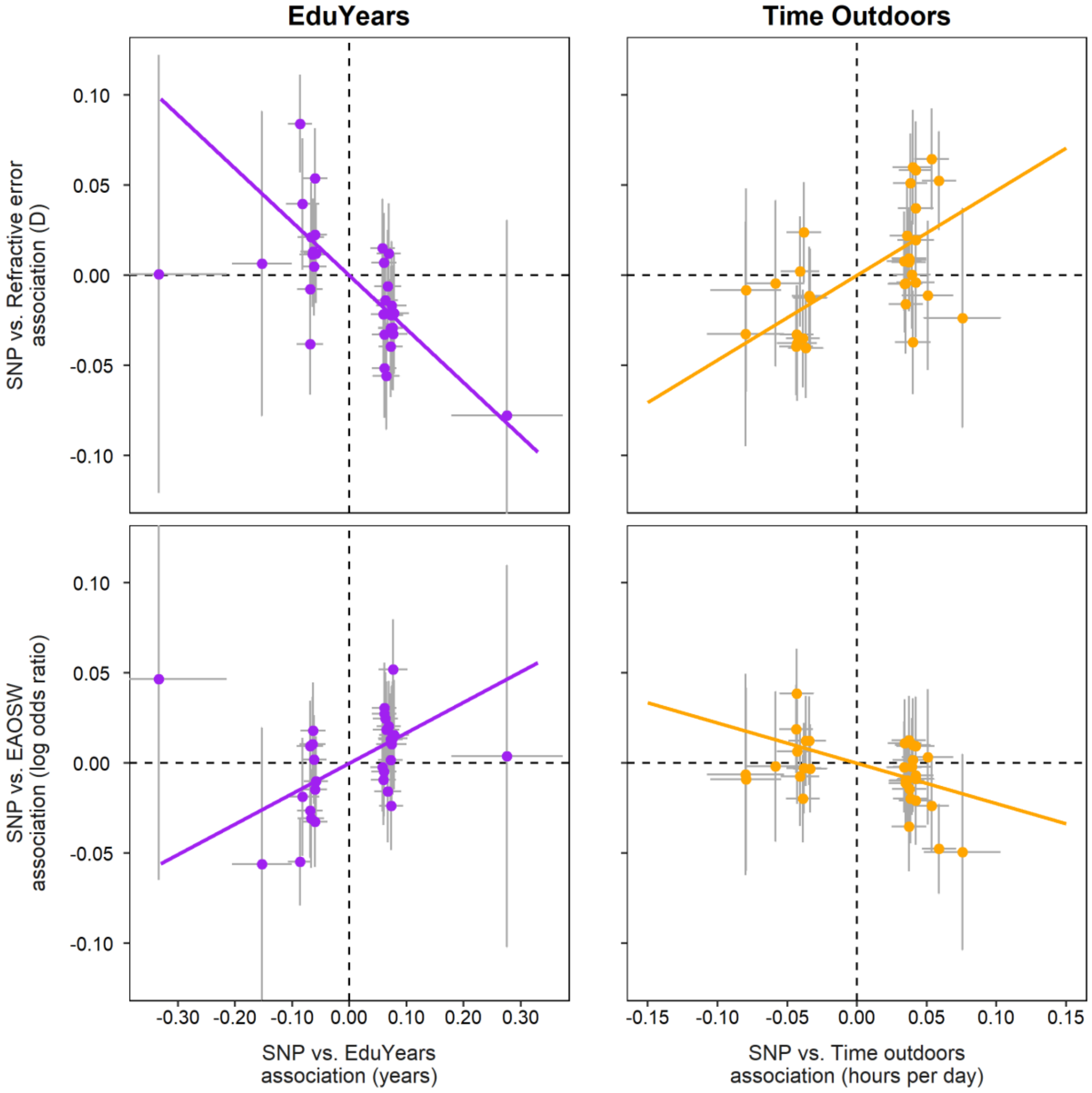

Figure S3. Graph of SNP-Exposure vs. SNP-Outcome for GWAS variants with P<1e-06. Univariable Mendelian randomisation a-lysis of the exposure EduYears or time spent outdoors on the outcome of either spherical equivalent refractive error or an EAOSW. Error bars show 95% CI. The slope of the solid line corresponds to the inverse-variance weighted Mendelian randomisation (MR-IVW) causal effect estimate.

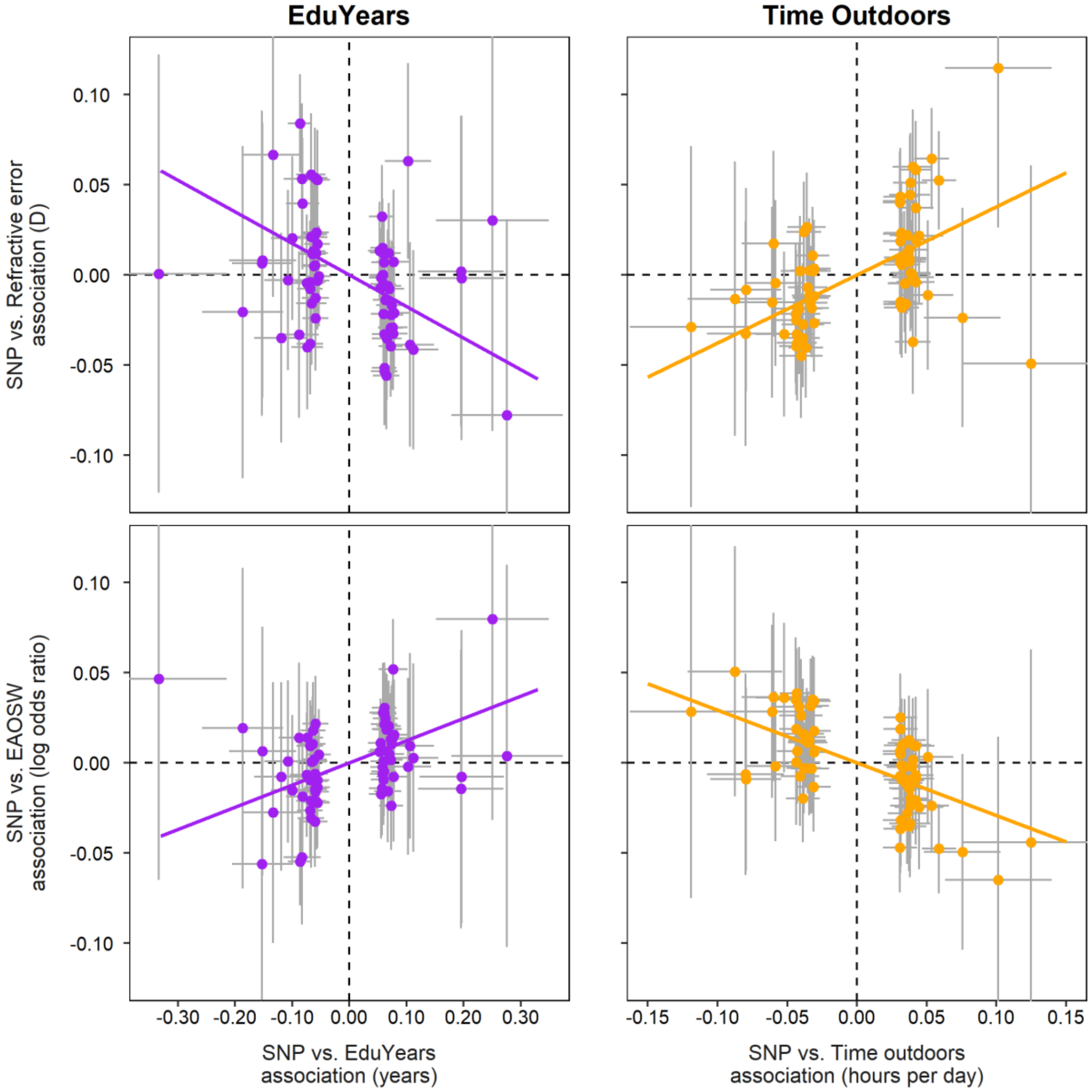

Figure S4. Comparison of univariable vs. multivariable MR a-lysis for variants with P<5e-08. Estimated causal effects of exposure to years spent in education (EduYears) and time spent outdoors on the outcomes (1) spherical equivalent refractive error, or (2) an early age-of-onset of spectacle wear (EAOSW). Error bars show 95% CI. Seven MR tests were evaluated: IVW = Inverse variance weighted MR and MVMR.; EGGER = MR-Egger and MVMR-Egger a-lysis; MEDIAN = weighted median MR and MVMR; MBE = mode-based estimate MR and MVMR; PRESSO = MR-PRESSO; ROBUST = robust MR and MVMR.

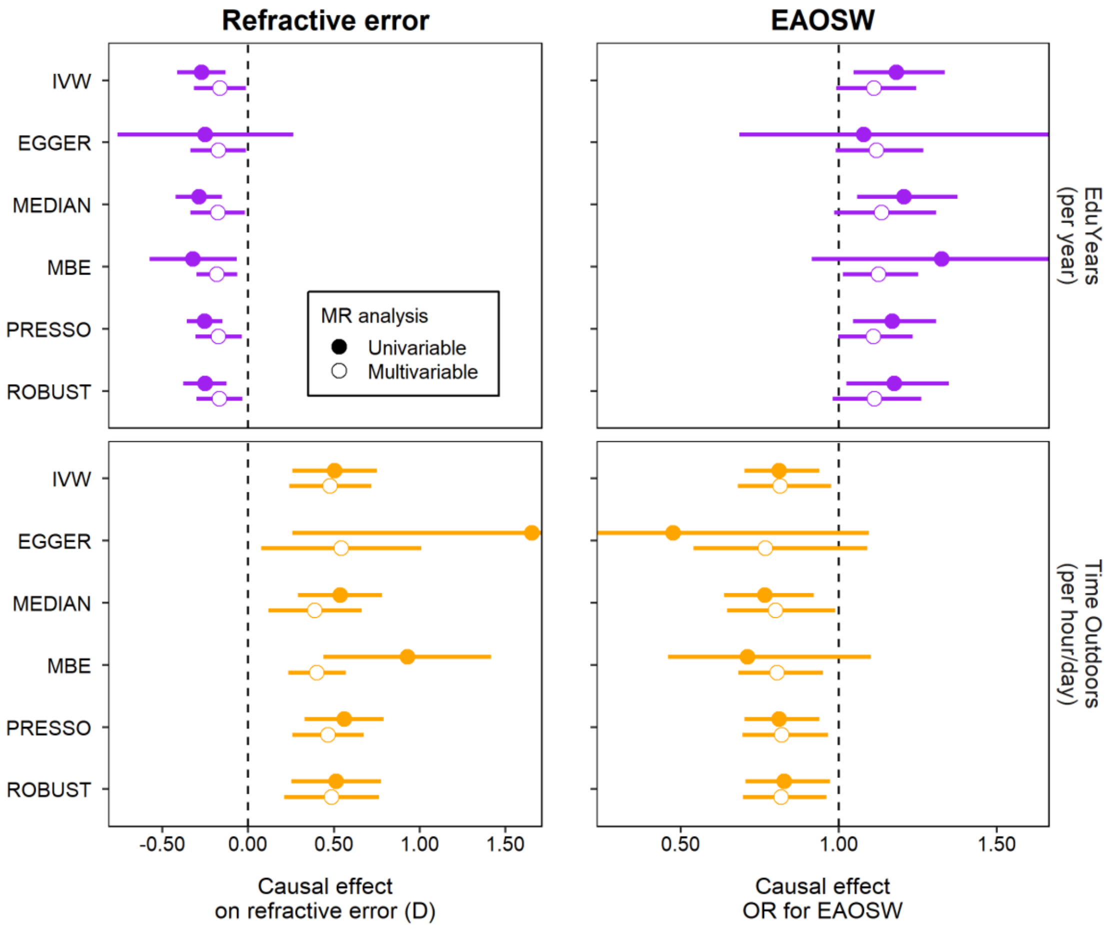

Figure S5. Comparison of univariable vs. multivariable MR a-lysis for variants with P<1e-07. Estimated causal effects of exposure to years spent in education (EduYears) and time spent outdoors on the outcomes (1) spherical equivalent refractive error, or (2) an early age-of-onset of spectacle wear (EAOSW). Error bars show 95% CI. Seven MR tests were evaluated: IVW = Inverse variance weighted MR and MVMR.; EGGER = MR-Egger and MVMR-Egger a-lysis; MEDIAN = weighted median MR and MVMR; MBE = mode-based estimate MR and MVMR; PRESSO = MR-PRESSO; ROBUST = robust MR and MVMR.

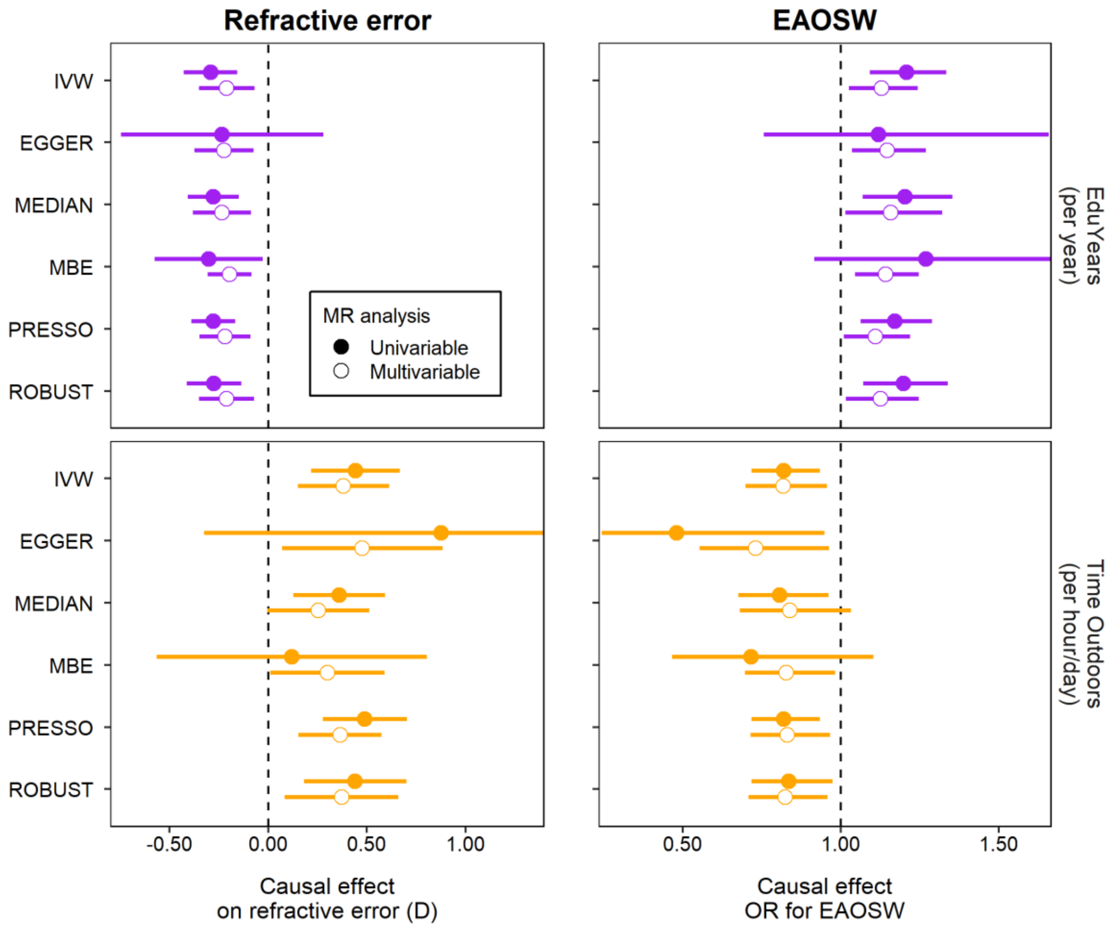

Figure S6. Comparison of univariable vs. multivariable MR a-lysis for variants with P<1e-06. Estimated causal effects of exposure to years spent in education (EduYears) and time spent outdoors on the outcomes (1) spherical equivalent refractive error, or (2) an early age-of-onset of spectacle wear (EAOSW). Error bars show 95% CI. Seven MR tests were evaluated: IVW = Inverse variance weighted MR and MVMR.; EGGER = MR-Egger and MVMR-Egger a-lysis; MEDIAN = weighted median MR and MVMR; MBE = mode-based estimate MR and MVMR; PRESSO = MR-PRESSO; ROBUST = robust MR and MVMR.

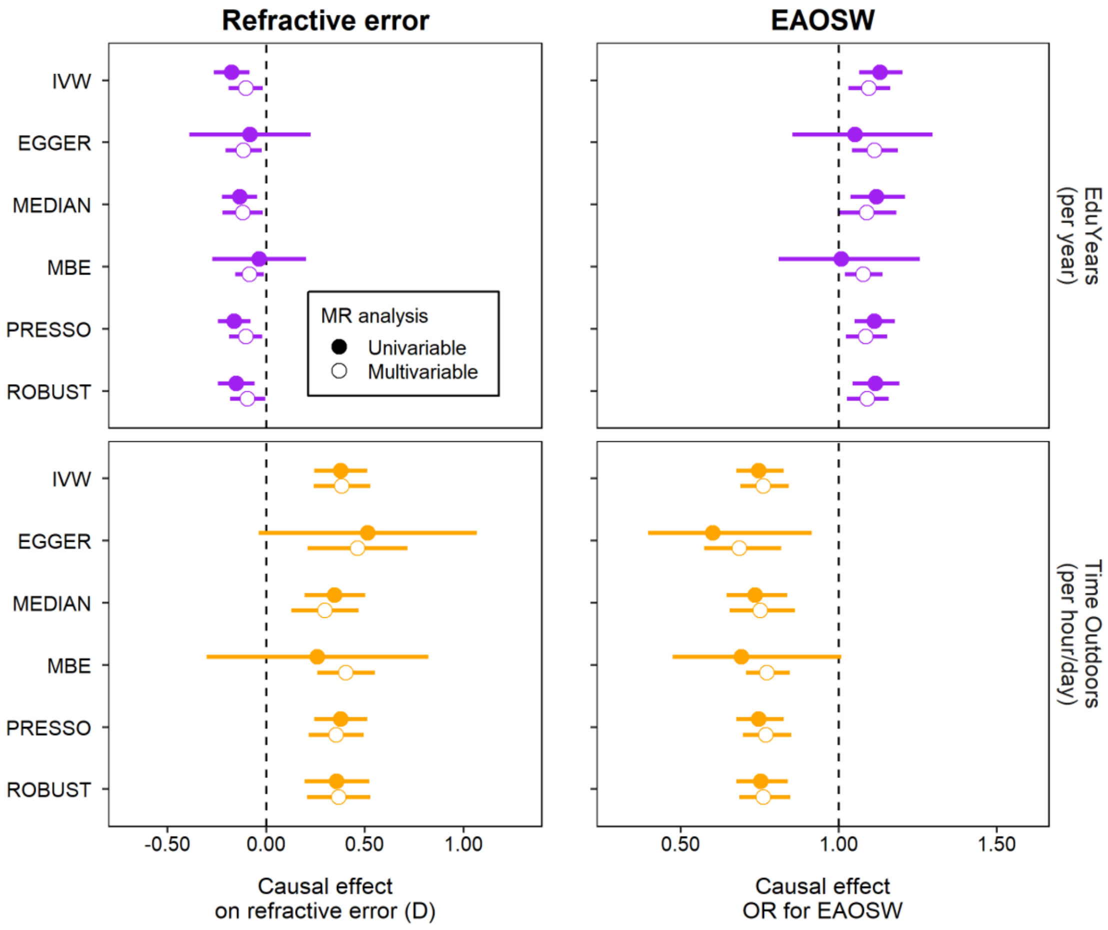

UK Biobank Eye and Vision Consortium members

-omi Allen, DPhil, University of Oxford; Tariq Aslam, MB BCh, DPhil, MD, The University of Manchester; Denize Atan, B.M,B.Ch(Oxon), MA(Cantab), PhD, University of Bristol; Konstantinos Balaskas, MD, MRCSEd, FRCOphth, Moorfields Eye Hospital; Sarah Barman, PhD, Kingston University; Jenny Barrett, PhD, University of Leeds; Paul Bishop, B Med Sci (Hons), BM BS, PhD, The University of Manchester; Graeme Black, MB BCh, DPhil, The University of Manchester; Tasanee Braithwaite, MB BCh, DM, MRCP, St Thomas' Hospital; Roxa- Carare, MD, PhD, University of Southampton; Usha Chakravarthy, MD, PhD, Queen's University Belfast; Michelle Chan, MBBS, Moorfields Eye Hospital; Sharon Chua, PhD, UCL Institute of Ophthalmology; Alexander Day, MBBS, PhD, Moorfields Eye Hospital; Parul Desai, PBM ChB PhD FRCOphth, Moorfields Eye Hospital; Bal Dhillon, BMedSci, BMBS, University of Edinburgh; Andrew Dick, M.B.B.S.(Lond.), M.D.(Aberd.), , University of Bristol; Alexander Doney, PhD, University of Dundee; Cathy Egan , MBBS , Moorfields Eye Hospital; Sarah Ennis, PhD, University of Southampton; Paul Foster, BMedSci(Hons), BM, BS, PhD, UCL Institute of Ophthalmology; Marcus Fruttiger, PhD, UCL Institute of Ophthalmology; John Gallacher, PhD, University of Oxford; David (Ted) Garway-Heath, MB ChB, UCL Institute of Ophthalmology; Jane Gibson, PhD, University of Southampton; Jeremy Guggenheim, PhD, Cardiff University; Chris Hammond, MA(Cantab), MB ChB, MD, King's College London; Alison Hardcastle, PhD, UCL Institute of Ophthalmology; Simon Harding, MB ChB, MD, University of Liverpool; Ruth Hogg, PhD, Queen's University Belfast; Pirro Hysi , MD, DPhil, King's College London; Pearse Keane, BMedSci, MD, UCL Institute of Ophthalmology; Sir Peng Tee Khaw, FMedSci, MB, PhD, FRCP, FRCPath, UCL Institute of Ophthalmology; Anthony Khawaja, MA(Cantab), MPhil, PhD, Moorfields Eye Hospital; Gerassimos Lascaratos, MBBS, PhD, Moorfields Eye Hospital; Thomas Littlejohns, PhD, University of Oxford; Andrew Lotery, MB BCh BAO, MD, University of Southampton; Robert Luben, PhD, UCL Institute of Ophthalmology; Phil Luthert, MBBS, PhD, UCL Institute of Ophthalmology; Tom MacGillivray , PhD, University of Edinburgh; Sarah Mackie, BM BCh, PhD, University of Leeds; Savita Madhusudhan, MBBS, DO, FRCOphth, Royal Liverpool University Hospital; Ber-dette McGuinness, MBBS, MD, PhD, Queen's University Belfast; Gareth McKay, PhD, Queen's University Belfast; Martin McKibbin , MBBS, Leeds Teaching Hospitals NHS Trust; Tony Moore, BM ChB FRCOphth MD, UCL Institute of Ophthalmology; James Morgan, DPhil, BM BCH, Cardiff University; Eoin O'Sullivan , MB ChB?, King's College Hospital; Richard Oram, BM BCh, PhD, University of Exeter; Chris Owen, PhD, St George's, University of London; Praveen Patel, MBBS, MD(Res), Moorfields Eye Hospital; Euan Paterson, BSc(Hon), PhD, Queen's University Belfast; Tunde Peto, PhD, Queen's University Belfast; Axel Petzold, MD, PhD, UCL Institute of Neurology; Nikolas Pontikos, M.Eng (hons), PhD, UCL Institute of Ophthalmology; Jugnoo Rahi , MBBS, PhD, UCL Institute of Child Health; Alicja Rudnicka , PhD, St George's, University of London; -veed Sattar, FMedSci FRCPath FRCPGlas FRSE, University of Glasgow; Jay Self, BM, PhD, University of Southampton; Pa-giotis Sergouniotis, MBBS, MD, PhD, The University of Manchester; Sobha Sivaprasad, MS Ophth, DM , Moorfields Eye Hospital; David Steel, MB ChB, MD, Newcastle University; Irene Stratton , MSc, Gloucestershire Hospitals NHS Foundation Trust; Nicholas Strouthidis, MBBS, MD, PhD, Moorfields Eye Hospital; Cathie Sudlow, MB, DPhil, University of Edinburgh; Zihan Sun, MBBS, MMed, PhD, UCL Institute of Ophthalmology; Robyn Tapp, PhD, St George's, University of London; Dhanes Thomas , MB ChB, MD, Moorfields Eye Hospital; Emanuele Trucco, PhD, University of Dundee; Ad-n Tufail, MBBS, MD, Moorfields Eye Hospital; A-nth Viswa-than, MD, PhD,

Moorfields Eye Hospital; Veronique Vitart, PhD, University of Edinburgh; Mike Weedon, PhD, University of Exeter; Katie Williams, MBChB, PhD, King's College London; Cathy Williams, MBBS, Ph.D, University of Bristol; Jayne Woodside, PhD, Queen's University Belfast; Max Yates, MBBS, PhD, University of East Anglia; Yalin Zheng, PhD, University of Liverpool.

.

Supplementary References

45. Guggenheim JA, Clark R, Zayats T, Williams C, UK Biobank Eye and Vision Consortium. Assessing the contribution of genetic nurture to refractive error. *Eur J Hum Genet.* 2022;30**:**1226-1232.
46. Bycroft C, Freeman C, Petkova D, et al. The UK Biobank resource with deep phenotyping and genomic data. *-ture.* 2018;562**:**203-209.
47. Loh P-R, Kichaev G, Gazal S, Schoech AP, Price AL. Mixed-model association for biobank-scale datasets. *-t Genet.* 2018;50**:**906-908.
48. Verhoeven VJM, Hysi PG, Wojciechowski R, et al. Genome-wide meta-a-lyses of multiancestry cohorts identify multiple new susceptibility loci for refractive error and myopia. *-t Genet.* 2013;45**:**314-318.
49. Tedja MS, Wojciechowski R, Hysi PG, et al. Genome-wide association meta-a-lysis highlights light-induced sig-ling as a driver for refractive error. *-t Genet.* 2018;50**:**834–848.
50. Hysi PG, Choquet H, Khawaja AP, et al. Meta-a-lysis of 542,934 subjects of European ancestry identifies new genes and mechanisms predisposing to refractive error and myopia. *-t Genet.* 2020;52**:**401-407.
51. Howe LJ, Nivard MG, Morris TT, et al. Within-sibship genome-wide association a-lyses decrease bias in estimates of direct genetic effects. *-t Genet.* 2022;54**:**581-592.
52. Privé F, Arbel J, Vilhjálmsson BJ. LDpred2: better, faster, stronger. *Bioinformatics.* 2020;36**:**5424-5431.
53. Chang CC, Chow CC, Tellier LC, et al. Second-generation PLINK: rising to the challenge of larger and richer datasets. *GigaScience.* 2015;4**:**7.
54. Hemani G, Zheng J, Elsworth B, et al. The MR-Base platform supports systematic causal inference across the human phenome. *Elife.* 2018;7**:**e3-08.
55. Broadbent JR, Foley CN, Grant AJ, et al. MendelianRandomization v0.5.0: updates to an R package for performing Mendelian randomization a-lyses using summarized data. *Wellcome Open Res.* 2020;5**:**252.
56. Verbanck M, Chen C-Y, Neale B, Do R. Detection of widespread horizontal pleiotropy in causal relationships inferred from Mendelian randomization between complex traits and diseases. *-t Genet.* 2018;50**:**693-698.
57. Grant AJ, Burgess S. Pleiotropy robust methods for multivariable Mendelian randomization. *Stat Med.* 2021;40**:**5813-5830.
58. Benjamin W, Dipender G, Andrew JG, Stephen B. MVMRmode: Introducing an R package for plurality valid estimators for multivariable Mendelian randomisation. *medRxiv.* 2023;2023.2001.2009.23284345.
59. Sanderson E, Spiller W, Bowden J. Testing and correcting for weak and pleiotropic instruments in two-sample multivariable Mendelian randomization. *Stat Med.* 2021;40**:**5434-5452.
60. Mountjoy E, Davies NM, Plotnikov D, et al. Education and myopia: assessing the direction of causality by mendelian randomisation. *BMJ.* 2018;361**:**k2022.
61. Plotnikov D, Sheehan -, Williams C, et al. Hyperopia Is Not Causally Associated With a Major Deficit in Educatio-l Attainment. *Transl Vis Sci Technol.* 2021;10**:**34.
